# Comparative insights into molecular pathways influencing germline development in early‐divergent angiosperms

**DOI:** 10.1111/tpj.70675

**Published:** 2026-01-18

**Authors:** Jorge Lora, Matthew R. Tucker, Neil J. Shirley, Chao Ma, José I. Hormaza

**Affiliations:** ^1^ Department of Subtropical Fruits Instituto de Hortofruticultura Subtropical y Mediterránea “La Mayora” (IHSM‐UMA‐CSIC) Algarrobo‐Costa Málaga 29750 Spain; ^2^ School of Agriculture, Food and Wine, Waite Research Institute The University of Adelaide Urrbrae South Australia 5064 Australia

**Keywords:** *Annona cherimola*, *Arabidopsis thaliana*, germline development, gametogenesis, magnoliids, ovule evolution, sporogenesis

## Abstract

The molecular and genetic mechanisms regulating female germline development remain largely unknown in basal and early‐divergent angiosperms. This contrasts with recent progress in model eudicots such as *Arabidopsis thaliana* and monocots, such as rice, barley, and maize. In this study, we investigate the genetic pathway governing female germline development in the early‐divergent angiosperm *Annona cherimola*. Using a region‐specific transcriptome study, key orthologs of germline regulators were identified, followed by spatio‐temporal expression pattern analysis and a functional assay. Expression patterns were compared to those in Arabidopsis. While some genes like *DMC1* show similar expression patterns across species, most genes display notable differences, though they are still transcribed in or around the germline. *AcWUS*, despite its differential expression compared to Arabidopsis, maintains its essential role in ovule and plant development. Our findings offer insight into the evolutionary conservation of pathways regulating germline development, highlighting both similarities and differences in the expression of key genes. These may stem from the phylogenetic distance and/or different nucellar morphologies between early‐divergent magnoliids and derived eudicots.

## Significance Statement

Understanding how reproductive developmental pathways are conserved or diverge is essential to tracing the evolution of angiosperm reproduction; however, the genetic regulation of female germline development remains poorly studied in early‐divergent angiosperms. By identifying conserved and divergent gene expression patterns in the early‐divergent angiosperm *Annona cherimola*, our study provides new insights into the evolutionary dynamics of germline regulatory networks across flowering plants.

## INTRODUCTION

In flowering plants, male and female germ cells originate directly from somatic cells within specialized reproductive structures. Male germline development takes place in the anther where, initially, a hypodermal archesporial cell divides periclinally to originate an inner primary sporogenous cell and an outer primary parietal cell that undergoes several mitotic divisions. While the sporogenous cells become microspore mother cells (MiMCs), the primary parietal cells become the somatic cell layers of the anther wall (binucleate tapetum, endothecium and epidermis). The MiMC undergoes meiosis to form a tetrad of haploid microspores (microsporogenesis), which then divide to produce two sperm cells (microgametogenesis) (McCormick, 1993).

Female germline development takes place within the ovule. In the ovule primordium, the diploid sporophytic cells undergo several mitotic divisions to give rise to the proximal funiculus, the central chalaza and the distal nucellus, which is surrounded by the integuments. In the nucellus, a single somatic cell differentiates and expands to become the so‐called archesporial cell that continues its expansion to become the megaspore mother cell (MeMC). The MeMC undergoes meiosis (megasporogenesis), producing four megaspores, of which only one develops to become the embryo sac (megagametogenesis) that generally contains seven cells, including the female gamete and the egg cell (Maheshwari, 1950).

The underlying molecular and genetic mechanisms that regulate male germline development have been intensively studied (Chang et al., 2011; Hackenberg & Twell, 2019; Russell & Jones, 2015; Saito et al., 2023; Vogler et al., 2017). More recently, increasing attention has been given toward understanding female germline development (Huang et al., 2023; Lora et al., 2019; Pinto et al., 2019). Female germline development has been mostly studied in the model eudicot *Arabidopsis thaliana* (Lieber et al., 2011; Lora et al., 2017, 2019; Lora & Hormaza, 2020; Qin et al., 2014) and model monocots such as rice (Hong, Tang, Shen, et al., 2012; Hong, Tang, Zhu, et al., 2012) and maize (Sheridan et al., 1996, 1999; Wang, Nan, et al., 2012). Research has revealed that epigenetic regulators, cell cycle genes, transcription factors, and mobile signaling molecules are involved in the expansion/growth of the primary germline cell but are inhibited in the surrounding somatic cells. Among these genes, a nuclear protein related to MADS box transcription factors, SPOROCYTELESS/NOZZLE (SPL/NZZ), is one of only a few proteins involved in both male and female germline specification (Schiefthaler et al., 1999; Yang et al., 1999). In the ovule, SPL/NZZ promotes the expression of the homeobox gene *WUSCHEL* (*WUS*) in the nucellus, which, in turn, is involved in the formation of the female germline by indirectly upregulating the small peptides WINDHOSE 1 (WIH1) and WIH2 (Gross‐Hardt et al., 2002; Lieber et al., 2011). The spatial domain of *SPL/NZZ* expression is regulated by *ARGONAUTE9* and *RNA DEPENDENT RNA POLYMERASE 6* (Mendes et al., 2020), components of small RNA pathways, to ensure MeMC identity is restricted to a single ovule cell.

Other regulators have been identified by specific transcriptome analysis of different ovule cells in Arabidopsis. The first transcriptional profile of the MeMC was generated by laser capture microdissection (LCM) in Arabidopsis, revealing an enrichment of transcriptional regulators and RNA helicases in the MeMC (Schmidt et al., 2011). The expression pattern of the RNA helicase, MNEM (MEM), was observed in the female germline and thought to be related to the restriction of the female germline to only one cell per ovule (Schmidt et al., 2011). Subsequent LCM studies reported an enrichment of other transcriptional regulators, including SPL and WUS, but in this case, in nucellus cells surrounding the MeMC (Tucker, Okada, Hu, et al., 2012). A third transcriptomic study that compared the transcriptome of *spl* mutants and wild‐type plants revealed the role of the cytochrome P450 monooxygenase KLUH (KLU)/CYP78A5 in female meiosis (Zhao et al., 2014). Later studies demonstrated that KLU has a role in the prevention of the acquisition of MeMC identity (Zhao et al., 2018). In Arabidopsis, a single‐cell transcriptome atlas of the MeMC and nucellus (Pinto et al., 2024) as well as ovule primordia have recently been constructed (Hou et al., 2021), while transcriptome analyses of ovule development have also been performed in other flowering plants, mainly in the model monocots rice (Kubo et al., 2013; Wu et al., 2015) and barley (Yang et al., 2025). In rice, a specific transcriptome study of the MeMC by LCM and a functional study of OsERECTA2 (OsER2) receptor‐like kinase revealed the role of OsER2 on female germline cell specification (Zhao et al., 2020). A similar role of the ERECTA family in female cell specification has also been recently reported in Arabidopsis (Cai et al., 2023; Huang et al., 2023). The rice ovule transcriptome has now been resolved with even greater clarity, as reported by Li et al. (2023) who used a single nucleus sequencing approach to uncover intriguing expression patterns in different domains of the developing rice ovule.

The relatively few advances in the study of the molecular and genetic mechanisms underlying the formation of germ cells in model plants contrasts with numerous morphological studies (Kelley & Gasser, 2009; Rudall, 2021). These have shown that morphological changes, such as a rapid expansion of the germ cell during microsporogenesis and megagametogenesis, are remarkably conserved in flowering plants (Endress, 2011; Gómez et al., 2015; McCormick, 1993). The location of the female germline is also highly conserved. In Arabidopsis, the MeMC develops in a specialized region of the nucellus with auxin and cytokinin gradients establishing a developmental axis in Arabidopsis (Lora et al., 2019), with auxin found distally in the epidermal nucellus cells and cytokinin found proximally (close to the chalaza) (Marsch‐Martínez et al., 2012; Schaller et al., 2015). Similar polarization has been reported in other flowering plants (Forestan et al., 2012; Lituiev et al., 2013; Tucker, Okada, Johnson, et al., 2012), including the early divergent species with crassinucellar ovules, *Annona cherimola* and *Persea americana* (members of the Magnoliid clade) (Lora et al., 2017), consistent with a conserved microenvironment during megasporogenesis. However, there are obvious differences between evolutionary derived and basal/early‐divergent angiosperms, for example in the nucellus, which surrounds the MeMC. Evolutionary derived angiosperms, such as Arabidopsis, show tenuinucellar ovules with a unicellular layer surrounding the MeMC, while basal and early divergent angiosperms, such as magnoliids, generally show crassinucellar ovules with several cell layers around the MeMC (Endress, 2011). These differences in morphology are accompanied by distinct gene expression patterns, suggesting that while the cellular microenvironment during germline development appears generally similar between angiosperms, there may be considerable mechanistic differences (Lora et al., 2017). Relatively few studies have considered the evolution of the microenvironment and the positional signal during megasporogenesis, mainly because little is known about the genetic mechanisms that regulate germline identity in non‐model plants. A better understanding of these mechanisms in basal and early divergent angiosperms could highlight evolutionary changes during germline development in derived eudicots.

To fill this gap, we continue previous studies of germline formation in *A. cherimola* (Lora et al., 2017), an early divergent angiosperm, via a detailed transcriptional analysis of young ovules and anthers. *A. cherimola* belongs to the Annonaceae, a family included within the order Magnoliales within the early divergent clade Magnoliid (APG IV, 2016) and with agronomic importance in subtropical climates. To evaluate genes expressed in the ovule and near the MeMC, we captured RNA using LCM and generated a transcriptome via RNA‐Seq. Using this database, we examined the expression of genes known to be involved in megasporogenesis in Arabidopsis (Lieber et al., 2011; Zhao et al., 2018) and validated their expression by *in situ* hybridization and RT‐qPCR. We also evaluated these genes in the early stages of anther development that show MiMC. Moreover, we performed a functional study of a potential *AcWUS* ortholog, using *Arabidopsis thaliana wus‐1* mutants, to test for complementation. Our findings provide new insights on the evolutionary conservation and divergence of genetic pathways regulating germline cell development and differentiation in angiosperms, shedding light on how these regulatory networks may have evolved from the early‐divergent magnoliids to derived eudicots.

## RESULTS

### Identification and expression profiling of candidate genes in *Annona cherimola* ovule development using LCM and *de novo* transcript assembly

To identify genes potentially involved in *A. cherimola* ovule development, we performed laser capture microdissection (LCM) on semi‐thin sections of young ovules. Three tissue pools were collected: (1) whole ovules containing the MeMC, (2) whole ovules where the MeMC was ablated, and (3) the nucellus region containing the MeMC (Figure S1). For comparison, LCM was also used to collect anther lobes containing MiMC, while whole fresh carpels, stamens, and young leaves were collected at the same stage to create a general transcript reference (Figure S2).

Given that the genome of *A. cherimola* was not available until recently (Talavera et al., 2023), a *de novo* transcript reference set was assembled in CLC genomics using previously published *Annona squamosa* RNA‐Seq data as a scaffold (SRA archive SRP074402 and Gupta et al. 2015). This resulted in the assembly of 122 552 contigs with an N50 of 1067 bp. The same contig set was used to guide the *de novo* assembly of *A. cherimola* contigs, using the RNA‐Seq data from the fresh carpel, anther and leaf samples. In total, 79 809 *A. cherimola* contigs were assembled, with an N50 of 1262 bp. Reciprocal best BLAST (RBB) analyses between *A. cherimola* contigs and the recently published *A. cherimola* genome showed that 38% of contigs produced RBBs with the genome, and 89.9% of genome transcripts recovered RBBs with contigs. This suggests that the *de novo* contig assembly may have given rise to multiple fragments from the same genome transcript, revealed different transcript isoforms and/or be due to low sequencing depth. Consistent with all of these possibilities, 80% of the 79 809 contigs mapped successfully to the genome. These results support the transcriptome assembly's reliability, but also potentially highlight the presence of contigs representing incomplete transcripts. To assess whether the contigs contain full length sequences representing previously characterized *A. cherimola* genes involved in ovule development, we searched for *INNER NO OUTER* (*INO*; At1g23420) and *PIN FORMED 1* (*PIN1*; *At1g73590*) (Lora et al., 2011, 2017). Full length coding sequences were identified for both genes (*AcINO* = contig_5628; *AcPIN1* = contig_432), suggesting that full length ovule sequences are present.

**Figure 1:**
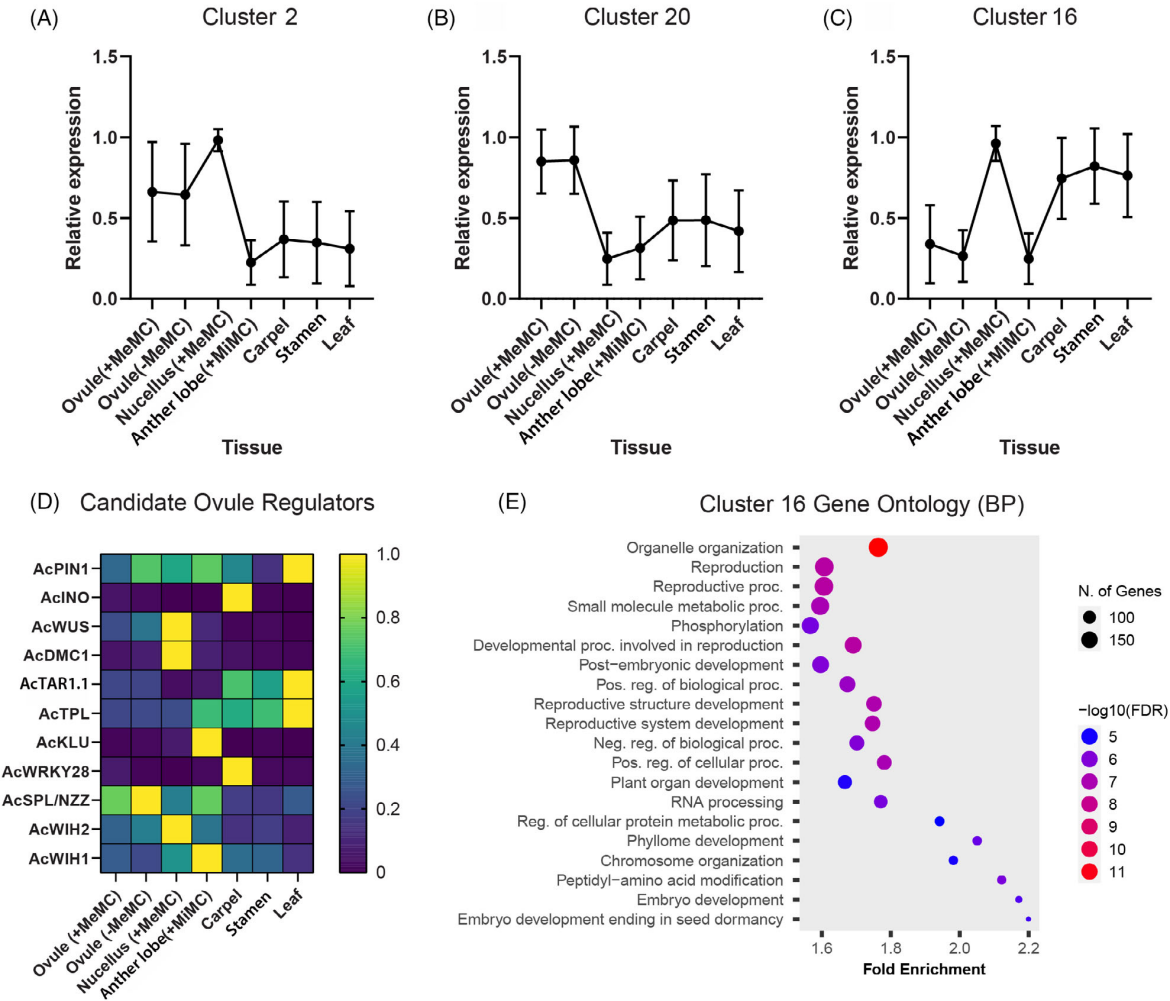
RNA‐Seq analysis of *A. cherimola* tissues collected by laser capture microdissection. (A–C) k‐means clustering revealed different patterns of gene expression dependent upon tissue types. Patterns were generated by normalizing TPM values to the maximum expression for each contig, and then clustered using k‐means clustering and Pearson correlations. Representative clusters showing prominent expression in ovule tissues are shown. (D) Expression pattern of selected contigs (normalized to the maximum expression value of each gene) showing homology to genes involved in Arabidopsis ovule development. (E) Gene ontology (biological process) analysis of 1828 contigs from Cluster 16. The homologous Arabidopsis gene identifier was used to assess enrichment and significance.

Next, we used the *A. cherimola* contigs as a reference to map all of the *A. cherimola* RNA‐Seq datasets. Normalized expression values (TPM) were generated for each contig, thereby providing a relative estimate of transcript abundance in different *A. cherimola* tissues. Around 82% of the contigs showed expression values >1 TPM, with an average maximum expression value of 42 TPM. Moreover, approximately 26% of the contigs were detected in the whole ovule LCM sample. Prominent expression patterns were determined using k‐means clustering, revealing distinct profiles for ovule (Cluster 2, 20; Figure 1A,B) and nucellus samples (Cluster 16; Figure 1C).

To test whether other candidate regulators of ovule development were present and expressed in *A. cherimola* ovules, we searched for genes such as *WUS*, *Disrupted Meiotic cDNA1* (*DMC1*), *TRYPTOPHAN AMINOTRANSFERASE OF ARABIDOPSIS1* (*TAA1*), *TOPLESS* (*TPL*), *KLUH*, *WRKY28*, *SPL*/*NZZ*, *WIH1*, and *WIH2*. Contigs were identified for all of these Arabidopsis candidates (Tables S1 and S2), and analysis revealed qualitative differences in their expression patterns (Figure 1D). For example, *AcWUS* (contig_27485) and *AcDMC1* (contig_25937) showed abundant expression in the nucellus LCM samples and both were grouped in Cluster 16. Gene ontology analysis of this cluster (containing 1828 contigs) was carried out using the homologous Arabidopsis gene identifiers in ShinyGO (Ge et al., 2020) and revealed a strong enrichment for genes involved in reproduction, organelle organization, and regulation of biological processes (Figure 1E).

Although these relative expression patterns and gene ontology terms suggest that key factors involved in *A. cherimola* ovule development have been captured, it is important to note that most of the transcriptomes (except for Ovule + MeMc and Carpel) are derived from a single pooled sample of many LCM sections. The pooling strategy was used to maximize chances of assembling representative full‐length contigs from the tissues of interest, rather than precisely defining gene expression profiles. Hence, further validation of interesting candidates was conducted by *in situ* hybridization and RT‐qPCR.

### Validation of predicted gene expression patterns in reproductive tissues

To validate and expand our understanding of genes involved in the regulation of *A. cherimola* germline development, we selected several key genes involved in Arabidopsis megasporogenesis (Lora et al., 2019; Pinto et al., 2019) that were previously identified in our transcriptome analysis. We confirmed the orthologous genes by phylogenetic analysis and their expression pattern by *in situ* hybridization and RT‐qPCR in ovules and anthers.

**Figure 2:**
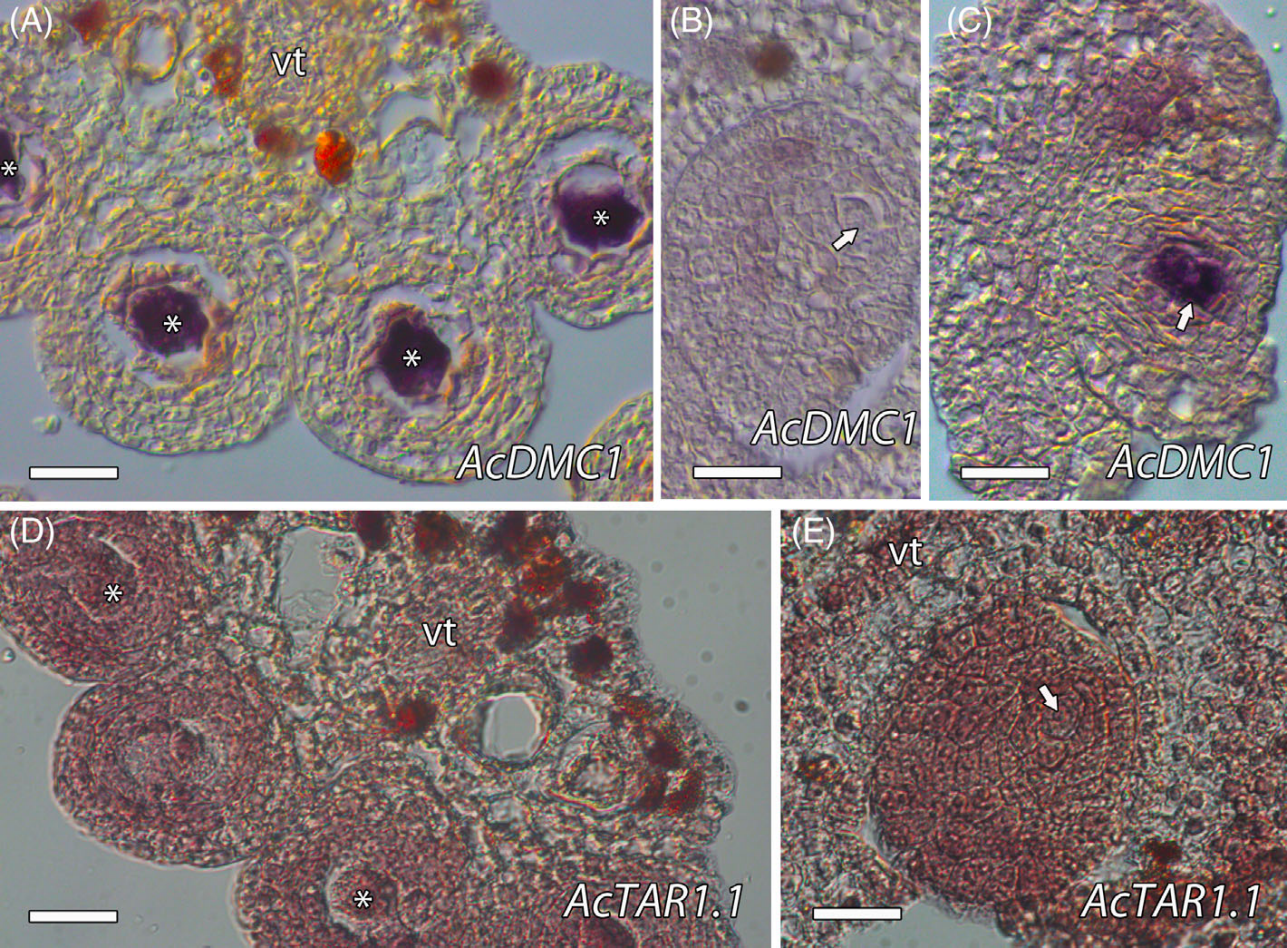
*In situ* hybridization of *AcDMC1* and *AcTAR1.1*. (A–C) *AcDMC1* expression was observed in the microspore mother cell (MiMC, asterisks) (A) and the megaspore mother cell (MeMC, arrows), but only just before meiosis (B, C). (D) *AcTAR1.1* was observed in the vascular tissue, MiMC, adjacent somatic cells, tapetum, and the outer layers of the anther wall. (E) In the ovule, *AcTAR1.1* expression was noted throughout the vascular tissue and the entire ovule, including the MeMC. vt, vascular tissue. Bars = 25 μm.

We first validated an essential gene involved in meiosis, *DMC1* (Klimyuk & Jones, 1997). The phylogenetic analysis showed that the predicted AcDMC1 (contig_25937/Anche102Chr3g0052200.1) was closely related to orthologs in other species (Figure S3). The expression of *AcDMC1* detected by *in situ* hybridization was only observed in MiMC of the anther (Figure 2A; Figure S4). In the ovule, expression was also observed in MeMC just before meiosis (Figure 2B,C; Figure S5).

In relation to the local microenvironment and phytohormones, we previously observed expression of the auxin efflux carrier *PIN1* distally in the nucellus and in the vascular tissue of *A. cherimola* ovules (Lora et al., 2017). Consistent with this, *AcPIN1* mRNA was also observed in the ovules. We further evaluated the auxin distribution by studying auxin biosynthetic genes, such as *TAA1* and its close homologs *TRYPTOPHAN AMINOTRANSFERASE RELATED1* (*TAR1*) and *2* (*TAR2*) (Matthes et al., 2019; Stepanova et al., 2008). From the RNA‐Seq data, we identified two relevant genes. Phylogenetic analysis placed one of these genes, which we named *AcTAR1.1* (contig_29342/Anche102Chr3g0054650.1), within the TAR1 subgroup of the TAA1/TAR family, in a lineage closely related to TAA1, which also includes TAA1 from *Amborella trichopoda*, a sister lineage to all extant angiosperms. The second gene, which we named *AcTAR3/4* (Anche102Chr4g0016960.1), grouped with the TAR3/TAR4 clade (Figure S6). We also found a third gene which we named *AcTAR1.2* (Anche102Chr3g0054630.1) in the genome of *A. cherimola* (Talavera et al., 2023), and that also grouped with the TAA1/TAR1/TAR2 clade. As *AcTAR1.1* was the only member of the TAA1/TAR1/TAR2 clade found in the RNA‐Seq data, we evaluated its expression by *in situ* hybridization. In the anthers, the expression of *AcTAR1.1* was observed in the vascular tissue and in the MiMC and the neighboring somatic cells, the tapetum and the outer layers of the anther wall (Figure 2D; Figure S4). In the ovule, the expression was also observed in the vascular tissue and in the whole ovule, including the MeMC (Figure 2E; Figure S5).

### Acquisition of MeMC identity

**Figure 3:**
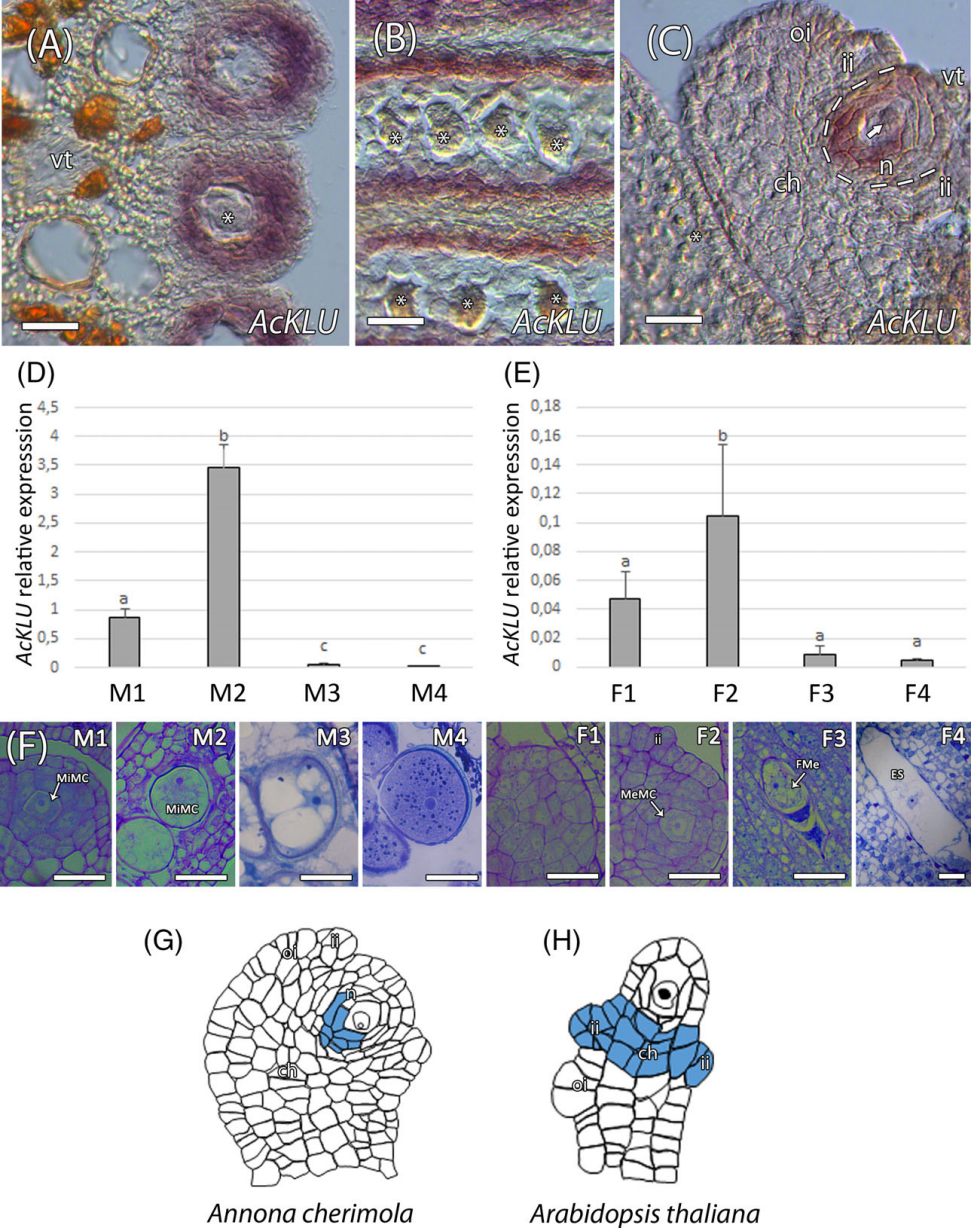
Evolutionary conservation of *AcKLU*. (A–C) *In situ* hybridization showing the expression pattern of *AcKLU*. (A, B) *AcKLU* expression was observed in the tapetum surrounding the microspore mother cell (MiMC, asteriks). (C) In the ovule, *AcKLU* was observed in the nucellus around the megaspore mother cell (MeMC, arrow). (D, E) Quantitative expression analysis of *AcKLU* by RT‐qPCR in four developmental stages in the anther (D) and ovule (E) with primary expression detected during the MiMC and MeMC developmental stages, respectively. Data represent mean ± SD of three biological replicates. Different letters above the bars indicate statistically significant differences among developmental stages (*P* ≤ 0.05; Duncan's multiple range test). (F) Four developmental stages of the anther (M1–M4) and the ovule (F1–F4): (M1) early microspore mother cell (MiMC), (M2) enlarged MiMC, (M3) vacuolated microspore before first mitosis, and (M4) mature pollen grain at anthesis, (F1) initial placental protrusion, (F2) megaspore mother cell (MeMC), (F3) functional megaspore (FMe) just after meiosis, and (F4) mature *Polygonum*‐type embryo sac. (G, H) Schematic representation of *KLU* expression in *A. cherimola* and Arabidopsis, showing localization in the nucellus near the MeMC (G) and in the chalaza and inner integument (H), respectively. ch, chalaza; ii, inner integument; n, nucellus; oi, outer integument. Bars = 25 μm.

We next evaluated the expression pattern of *AcKLU*, which is an essential gene involved in preventing the acquisition of MeMC‐like identity in the neighboring somatic cells in the ovules of Arabidopsis (Zhao et al., 2018). Phylogenetic analysis showed that the predicted AcKLU (contig_5911/Anche102Chr2g0050990.1) clustered within a clade exclusively containing KLU orthologs (Figure S7). The expression of *AcKLU* was only observed around the MiMC, in the tapetum and in the nucellus adjacent to the chalaza and around the MeMC (Figure 3A–C; Figure S5). We further analyzed the expression of *AcKLU* by RT‐qPCR in four developmental stages of anther and ovule (Figure 3D–F). *AcKLU* expression was only observed during the early developmental stages, slightly in the early MiMC and in the ovule primordium and mostly when MiMC and MeMC were apparent in the anther and ovule, respectively (Figure 3D,E).

### Formation of the MiMC and MeMC

**Figure 4:**
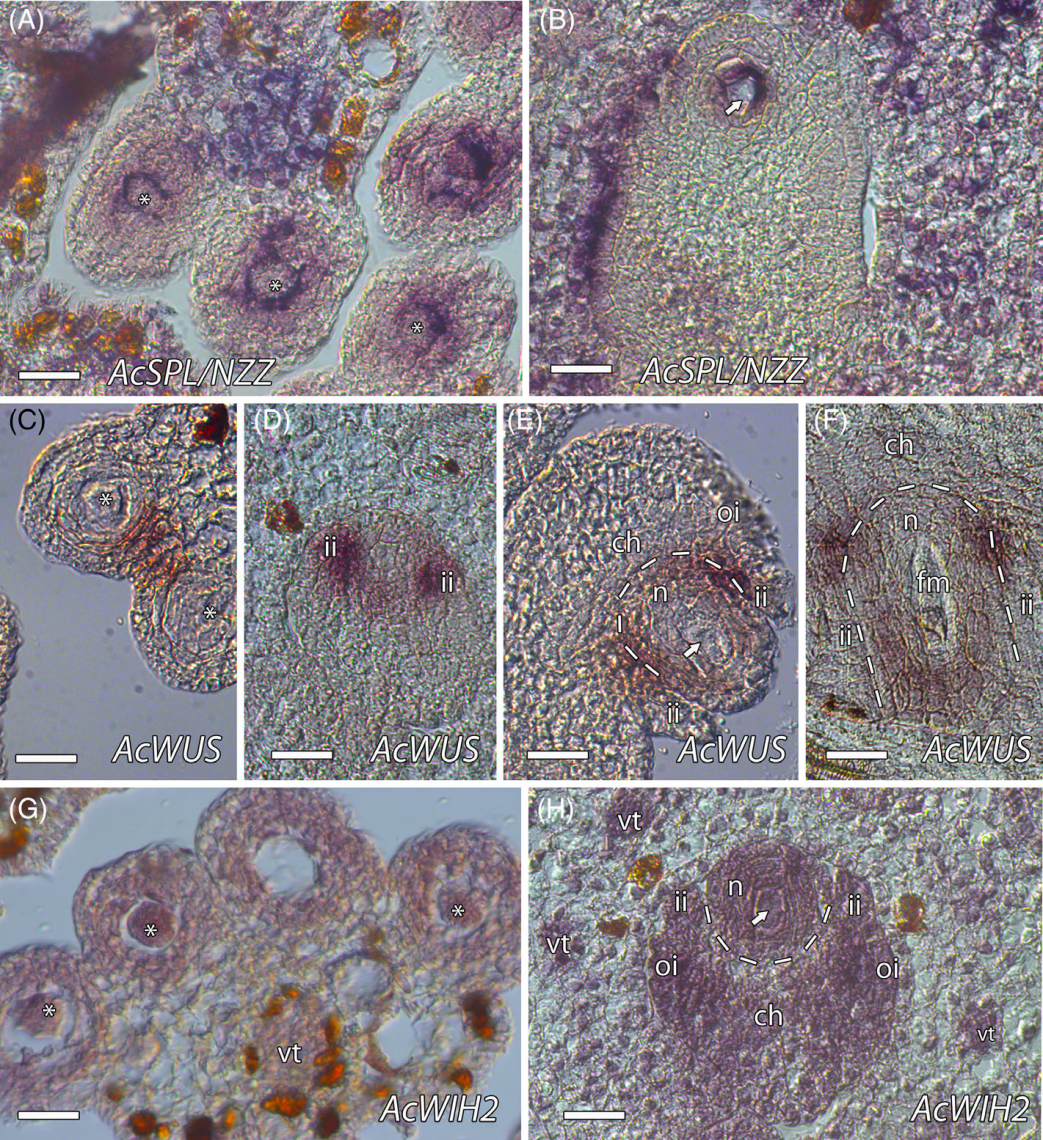
*In situ* hybridization of *AcSPL*/*NZZ*, *AcWUS*, and *AcWIH2*. (A. B) *AcSPL*/*NZZ* expression was observed around the microspore mother cell (MiMC, indicated by asterisks) in (A) and around the megaspore mother cell (MeMC, indicated by an arrow) in (B). (C) *AcWUS* expression in the anther was localized to the stomium region. (D) During early ovule developmental stages when the MeMC is incipient, *AcWUS* was primarily observed around the nucellus and in the inner integument. (E) *AcWUS* expression persisted as the MeMC became more apparent, with a slight extension into the basal boundary between the inner integument and nucellus. (F) During meiosis, *AcWUS* expression also extended slightly toward the distal pole of the nucellus. (G) *AcWIH2* expression was observed in the anther wall and in the MiMC. (H) In the ovule, *AcWIH2* was detected in the vascular tissue, chalaza, integuments, nucellus, and around the MeMC, but was notably absent around the nucellus. ch, chalaza; ii, inner integument; n, nucellus; oi, outer integument. Bars = 25 μm.

To analyze the initiation of the germ cells, we examined *SPL/NZZ*, *WUS*, and *WIH* that have been identified in the same genetic pathway promoting the transition from somatic to reproductive cell fate in Arabidopsis (Lieber et al., 2011). We also included TPL that interacts with SPL/NZZ (Chen et al., 2014; Wei et al., 2015). The predicted AcSPL/NZZ (contig_34527/Anche102Chr5g0016450.1), AcWUS (contig_27485/Anche102Chr6g0057050.1), AcWIH (AcWIH1, contig_2990/Anche102Chr6g0089940.1; AcWIH2, contig_7985/Anche102Chr4g0051720.1), and AcTPL (contig_379/Anche102Chr2g0056280.2) identities were supported by phylogenetic analysis that showed clades exclusively containing SPL/NZZ, WUS, WIH, and TPL orthologs, respectively (Figures S8–S11). The expression pattern of *AcSPL/NZZ* was observed in the cells adjacent to the MiMC and MeMC in the anther and ovule, respectively. Expression was also observed in the connective tissue of the anther and the ovary wall (Figure 4A,B; Figures S4 and S5). The expression of *AcWUS* was found in the stomium region of the anther, with a weaker expression around the MeMC in the ovule. Instead, expression was more prominent around the nucellus in the chalaza and the inner integument. In early stages of ovule development, *AcWUS* was expressed during the initiation of the inner integument and the incipient MeMC. This expression pattern continued during the expansion of the MeMC, and it was slightly extended at the base of the boundary between the inner integument and the nucellus (Figure 4C–F; Figures S4 and S5). During meiosis, *AcWUS* was also weakly observed at the distal pole of the nucellus (Figure 4F).

Phylogenetic analysis of WIH orthologs revealed two WIH‐like sequences that grouped into the WIH clade, but could not be directly assigned to WIH1, WIH2 or WIH3 protein identity from Arabidopsis (Figure S10). These sequences were arbitrarily named *AcWIH1* and *AcWIH2*, and the expression pattern of *AcWIH2* was evaluated by *in situ* hybridization. *AcWIH2* was expressed in the vascular tissue and in the MiMC, the tapetum and layers of anther wall. In the ovule, *AcWIH2* was expressed in the vascular tissue, the MeMC and the nucellus and in the integuments and part of the chalaza but, interestingly, not around the basal region of the nucellus where *AcWUS* was observed (Figure 4G,H; Figures S4 and S5).

**Figure 5:**
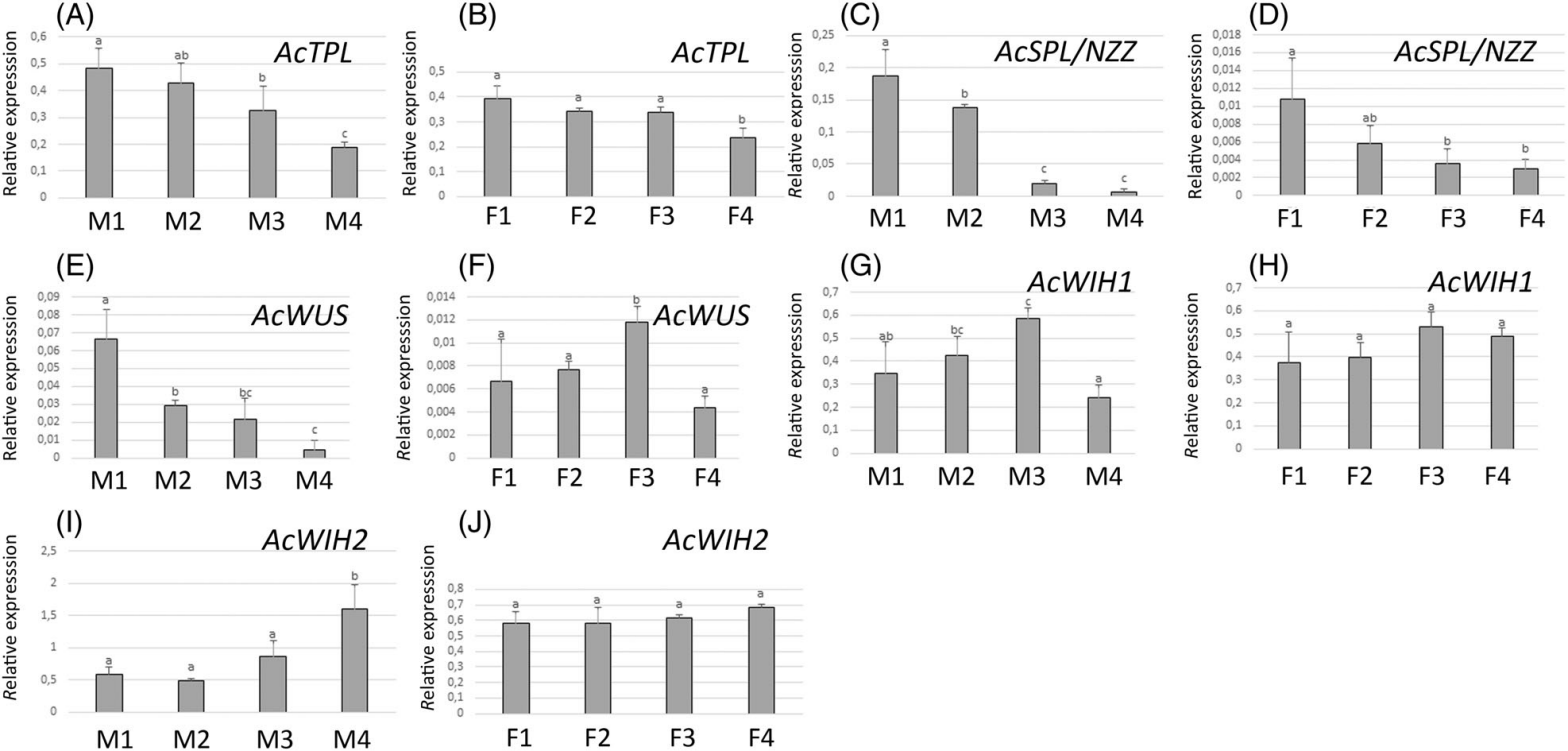
RT‐qPCR expression analysis of *AcTPL* (A, B), *AcSPL/NZZ* (C, D), *AcWUS* (E, F), *AcWIH1* (G, H), and *AcWIH2* (I, J) across four developmental stages in the anther (A, C, E, G, I) and ovule (B, D, F, H, J) (see also Figure 3F). (A, B) *AcTPL* showed the highest expression at early developmental stages, decreasing gradually with the lowest expression at anthesis in both anther (A) and ovule (B). (C, D) *AcSPL/NZZ* was highly expressed during early developmental stages in both the anther (early MiMC stage) (C) and the ovule primordium (D). (E, F) *AcWUS* was primarily expressed at stage M1, corresponding to the early MiMC stage in the anther (E), while its expression in the ovule was significantly higher during meiosis (stage F3) (F). (G, H) *AcWIH1* expression was lower at anther dehiscence (G), with no significant differences observed across the four developmental stages in the ovule (H). (I, J) *AcWIH2* expression varied significantly at anther dehiscence (I) but showed similar levels across the four ovule developmental stages (J). Data represent mean ± SD of three biological replicates. Different letters above the bars indicate statistically significant differences among developmental stages (*P* ≤ 0.05; Duncan's multiple range test).

In the case of *TPL*, we found two similar genes (Contig_379/Anche102Chr2g0056280.2 and contig_17379/Anche102Chr2g0026420.1) in the clade containing TPL orthologs (Figure S11). We named them *AcTPL1.1* and *AcTPL1.2* respectively. We evaluated the expression pattern of *AcTPL1.2* by RT‐qPCR in four stages of anther and ovule development from the earliest MiMC developmental stage and ovule primordium until anthesis. The highest expression levels were observed during early stages and meiosis, with reduced expression at anthesis (Figure 5A,B). The expression patterns of *AcSPL/NZZ*, *AcWUS*, and *AcWIH1/2* were also analyzed using RT‐qPCR. *AcSPL/NZZ* expression was confirmed during early developmental stages prior to meiosis in both the anther and ovule, with expression observed as early as the MiMC development stage and ovule primordium development (Figure 5C,D). *AcWUS* showed high expression during the earliest stages of MiMC development in the anther and decreased during later stages of MiMC development and the tetrad stage, with weak expression observed at anthesis. In the ovule, *AcWUS* expression was highest during meiosis and significantly reduced by anthesis (Figure 5E,F). For *AcWIH*, we evaluated the two *AcWIH* orthologs. In the anther, the highest expression of *AcWIH1* was observed at meiosis, while *AcWIH2* expression was highly significant at anthesis. In the ovule, *AcWIH1* and *AcWIH2* expression was not significantly different from the ovule primordium stage until anthesis (Figure 5G–J).

**Figure 6:**
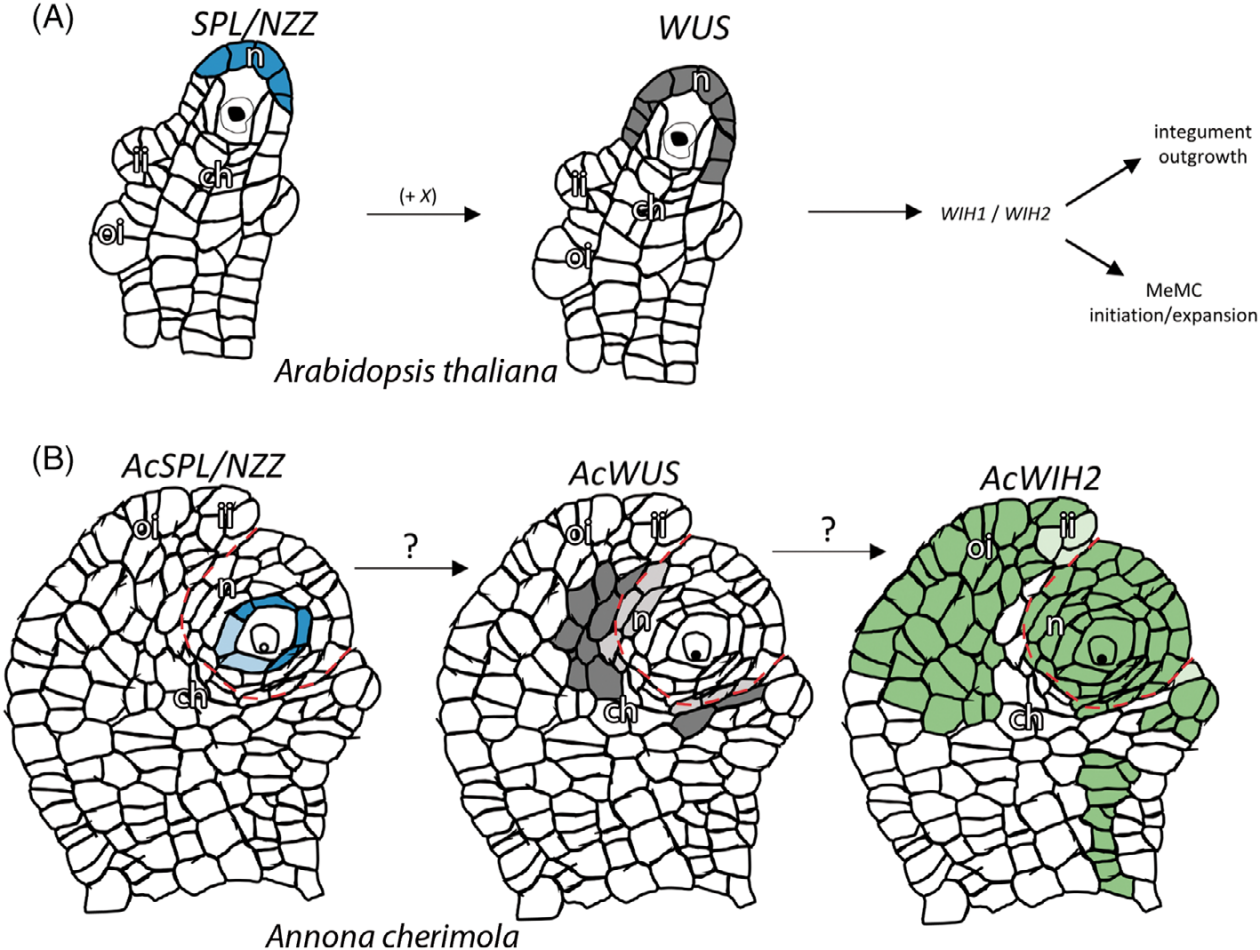
Scheme of the expression pattern of *SPL/NZZ* and *WUS* in *Arabidopsis thaliana* (A), and *AcSPL/NZZ*, *AcWUS*, and *AcWIH2* in *Annona cherimola* (B). (A) In Arabidopsis, *SPL/NZZ* is expressed distally in the nucellus near the megaspore mother cell (MeMC). *WUS* is expressed distally in the nucellus, overlapping with *NZZ* expression. (B) In *A. cherimola*, *AcSPL/NZZ* is expressed around the MeMC. *AcWUS* is primarily observed proximally around the nucellus in the basal boundary between the inner integument and nucellus. *AcWIH2* is mainly observed in the integuments, nucellus, and in the MeMC, but does not appear to overlap with *AcWUS* expression. ch, chalaza; ii, inner integument; n, nucellus; oi, outer integument.

Overall, these expression studies revealed a similar temporal pattern of expression to some of their orthologous genes in Arabidopsis such as *SPL/NZZ*, *TPL*, and *WUS*. However, in *A. cherimola*, the tissue‐specific expression patterns showed notable differences, including the expression of *AcSPL* surrounding the MeMC and the proximal expression of *AcWUS* around the nucellus (Figures 4, 5, 6).

### Functional study of *AcWUS*

**Figure 7:**
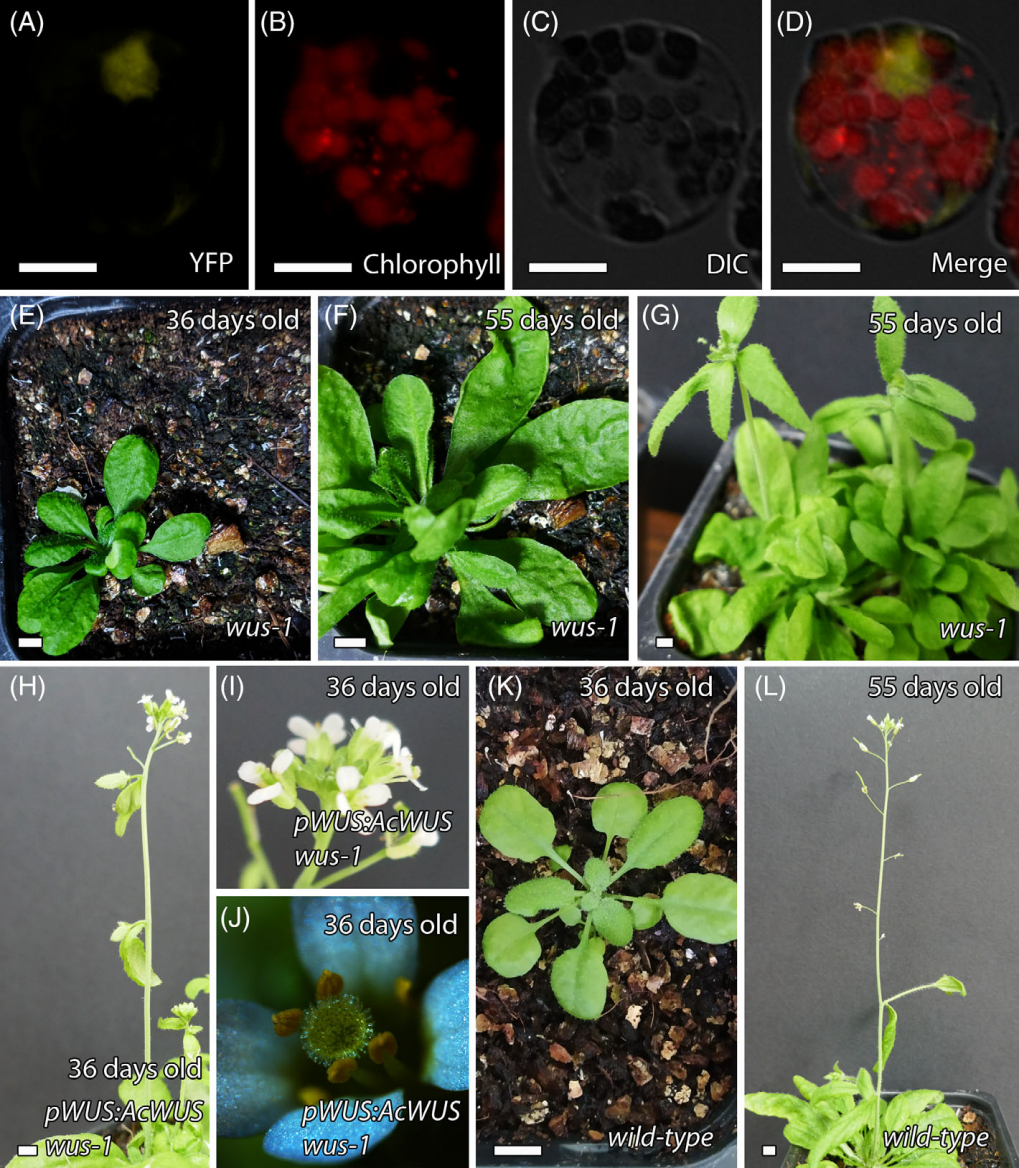
Functional study of *AcWUS*. (A–D) Nuclear expression of *AcWUS* visualized using *35S:AcWUS‐YFP* in cherimoya protoplast. Merged images are shown with YFP, diferential interference contrast (DIC) and chlorophyll autofluorescence. (E–G) *wus‐1* mutant at 36 days (E) and 55 days (G), showing loss of meristem integrity with disorganized clusters of leaves. (H–J) *wus‐1* mutant rescued by *pWUS:AcWUS* transgene, displaying a wild‐type phenotype at 36 days. (K, L) Wild‐type plants at 36 days (K) and 55 days (L). Rescue was confirmed across generations: 8 of 18 T2 and all 21 T3 transgenic lines carrying *pWUS:AcWUS* in a *wus‐1* homozygous background displayed a restored wild‐type phenotype, including inflorescence formation and seed set (see Table S3). Bars: (A–D) 10 μm; (E–L) 50 mm.

To further investigate the function of *AcWUS*, we first confirmed its nuclear localization by transiently expressing *35S:AcWUS‐YFP* in *A. cherimola* protoplasts (Figure 7A–D; Figure S12), a technique applied here for the first time in Annonaceae. We next performed cross‐species complementation on the *wus‐1* mutant, using *AcWUS* that was driven by the native *WUS* promoter of Arabidopsis. In Arabidopsis, *WUS* plays a central role in diverse signaling pathways related to plant growth and development, including megasporogenesis (Holt et al., 2014; Lieber et al., 2011). The *wus‐1* mutant fails to initiate normal rosette leaves and, later, the inflorescences, instead showing desorganized bunches of leaves (Laux et al., 1996) (Figure 7E–G). Using a selectable medium containing norflurazon, we obtained multiple independent T1 transgenic lines, two of which exhibited a *wus‐1* heterozygous genotype. From these two T1 transgenic lines, we obtained 18 T2 transgenic lines, of which 8 exhibited a *wus‐1* homozygous genotype but wild‐type phenotype with inflorescences. Six of the T2 transgenic lines homozygous for *wus‐1* produced seeds, four of which were followed to the T3 generation. The study of 21 T3 transgenic lines showed that all had a *wus‐1* homozygous genotype and produced inflorescences. Most of the T3 trangenic lines also produced seeds ranging from 0.1 ± 0.31 seeds/silique to 34 ± 5 seeds/silique (*n* ≥ 10 siliques) (Table S3; Figure 7H–L).

To further characterize reproductive development in the complemented lines, we analyzed anther dehiscence and ovule formation. Anther dehiscence was partial, with 15–44% of anthers releasing pollen. Ovule development was also variable, with ovule numbers ranging from 4 to 61 per gynoecium, although most gynoecia contained approximately 35–40 ovules. In some cases, gynoecia lacked ovules entirely, and the pistil was absent (Table S4; Figure S13). These results indicate that *AcWUS* is capable of partially rescuing the *wus‐1* phenotype, affecting both male and female reproductive structures. This suggests functional conservation of the orthologous *WUS* gene in the early‐divergent angiosperm *A. cherimola*, with potential divergence in regulatory or interaction contexts.

## DISCUSSION

### Preventing the acquisition of MeMC identity

*A. cherimola* shows crassinucellate ovules in which the female germline develops surrounded by multiple layers of somatic cells (nucellus), an ancestral feature shared with other basal angiosperms and extant gymnosperms (Endress, 2011; Fiordi, 1987; Takaso & Bouman, 1986). This ancestral trait contrasts with the tenuinucellate ovules of *Arabidopsis thaliana* and other derived angiosperms, in which the female germline is surrounded by a single nucellar layer (Endress, 2011). In both types of ovules, however, only one nucellar cell expands to become the MeMC, while MeMC fate is blocked in surrounding somatic cells.

The molecular mechanism restricting MeMC fate has been primarily studied in Arabidopsis. It has been suggested that MeMC fate and its expansion may be restricted by epigenetic modifications (Armenta‐Medina et al., 2011; Mendes et al., 2020; Olmedo‐Monfil et al., 2010; Su et al., 2017). Specifically, the enzyme KLU, a cytochrome P450 monooxygenase (CYP78A5), plays a pivotal role inhibiting MeMC fate in a non‐cell‐autonomous manner by activating WRKY28 through epigenetic H2A.Z deposition on *WRKY28* (Zhao et al., 2018). KLU, which is highly conserved in land plants (Zhao et al., 2016), has been mainly studied in Arabidopsis, where it promotes cell proliferation in a non‐cell‐autonomous manner (Anastasiou et al., 2007). Given its role in cell proliferation, studies on KLU homologs in crop plants have primarily focused on their effects on fruit weight and size (Guo et al., 2022; Li et al., 2021, 2022; Qi et al., 2023). However, to our knowledge, the role of KLU homologs in female germline development in other angiosperms remains underexplored, with exceptions such as recent expression studies in tomato (Gupta et al., 2021) and pineapple (Zhao et al., 2021), although spatial expression data is lacking. In Arabidopsis ovules, *KLU* expression has been observed at the base of the nucellus and in the inner integument (Adamski et al., 2009). In *A. cherimola*, *AcKLU* expression is also observed in the proximal region of the ovule, but in this case, in nucellar cells near the MeMC as well as in tapetal cells surrounding the MiMC in the anther. Interestingly, *AcKLU* expression is only observed during the formation of the MeMC and MiMC, suggesting a temporally regulated role in early germline cell differentiation.

### The acquisition of germline identity

During the earliest developmental stages, before the formation of MiMC and MeMC and their specific niches, the cells inside the anther and ovule appear morphologically very similar. *SPL/NZZ* is one of the few known genes involved in the formation of both male and female germline cells (Lora & Hormaza, 2020; Schiefthaler et al., 1999; Yang et al., 1999). In Arabidopsis, *SPL/NZZ* expression has been observed in the ovule primordium and later in the MeMC, the developing integuments (Balasubramanian & Schneitz, 2000; Schiefthaler et al., 1999), and the nucellus (Bencivenga et al., 2012). More recently, a functional SPL/NZZ‐GFP fusion protein was observed distally in the nucellus (L1 layer), suggesting a non‐cell‐autonomous regulation of MeMC identity by SPL/NZZ (Mendes et al., 2020). The underlying molecular mechanism regulated by *SPL/NZZ* has only been studied in Arabidopsis, in which SPL/NZZ interacts with TPL/TPR corepressors to inhibit the activity of CINCINNATA (CIN)‐like TEOSINTE BRANCHED1/CYCLOIDEA/PCF (TCP) transcription factors (Chen et al., 2014; Wei et al., 2015).

While *SPL/NZZ* orthologs remain largely unexplored in angiosperms, phylogenetic analyses indicate that they are broadly distributed in land plants (Chen et al., 2014). In tomato, the *SPL/NZZ* ortholog *HYDRA* is expressed in germline cells and in the nucellus of its tenuinucellate ovule, with similar expression patterns observed in the crassinucellate ovule of cucumber (Ren et al., 2018; Rojas‐Gracia et al., 2017). In *A. cherimola*, *SPL/NZZ* expression is observed in a unicellular layer of nucellus surrounding both the MiMC and MeMC, consistent with its dual role in the formation of both MiMC and MeMC in Arabidopsis (Schiefthaler et al., 1999; Yang et al., 1999). Despite differences in nucellar layers, *SPL/NZZ* and *AcSPL/NZZ* consistently localize near the MeMC, suggesting a conserved role in germline formation across species with distinct ovule architectures.

During the initiation of the MeMC in Arabidopsis, *SPL/NZZ* acts upstream of *WUS* (Lieber et al., 2011). While WUS does not appear to be involved in male germline formation, it plays a role in anther deshiscence (Deyhle et al., 2007). In Arabidopsis, *WUS* is expressed in the stomium region (Deyhle et al., 2007), similar to the expression of *AcWUS* in the anther of *A. cherimola*.

### *AcWUS* partially complements Arabidopsis *wus‐1*, indicating conservation of core WUS function despite expression differences

The role of WUS in the ovule has been studied in relatively few angiosperms. Examples include recent evaluations of *WUS* expression by a transcriptome study on early ovule developmental stages in pineapple (Zhao et al., 2021), by *in situ* hybridization in cucumber (Liu et al., 2018), and by quantitative expression in *Vitis vinifera* (Zhang et al., 2023). In tomato, silencing *WUS* through RNA interference revealed a role in flower development (Li et al., 2017), while phylogenetic analyses have identified *WUS* homologs across many angiosperms, including basal angiosperms such as *Amborella trichopoda* (Nardmann et al., 2009). Homologs of *WUS* have also been found in gymnosperms (Alvarez et al., 2018; Hedman et al., 2013), including a single *WUS/WOX5* homolog (Alvarez et al., 2018; Nardmann et al., 2009; Nardmann & Werr, 2013). Most functional studies have focused on the role of WUS in shoot meristem maintenance (Kieffer et al., 2006; Tanaka et al., 2015) and have mainly relied on overexpression assays to analyze effects (Li et al., 2020; Liu et al., 2014; Wang, Wang, et al., 2012; Yang et al., 2019). These assays have also been used to induce somatic embryogenesis in several angiosperms (Jha et al., 2020), including crops where it has been applied to improve agronomic traits (Du et al., 2025) and in the early‐divergent angiosperm *Ocotea catharinensis* (Santa‐Catarina et al., 2012).

From an evolutionary perspective, cross‐species complementation studies remain limited. Zhang et al. (2017) demonstrated that *WUS/WOX5* homologs from angiosperms, including *Amborella trichopoda*, and gymnosperms can almost completely rescue Arabidopsis *wus‐1* and *wox5‐1* mutants when expressed under the native promoter, whereas the fern homolog showed only partial rescue. In contrast, overexpression of *TaWUS* from wheat (Li et al., 2020) or *SlWUS* from tomato (Wang, Wang, et al., 2012) in Arabidopsis failed to fully replicate the effects of native *WUS* overexpression, suggesting that differences in regulatory context or protein interactions can limit functional equivalence. Our results align with this view. The finding that *AcWUS* partially rescues the *wus‐1* mutant phenotype – restoring inflorescence formation but not fully recovering fertility – suggests that while core WUSCHEL functions are conserved, some aspects of its role in reproductive development may have diverged. These findings highlight that, although WUS function in meristem maintenance and ovule development is broadly conserved, lineage‐specific modifications may influence its efficiency in heterologous systems.

In Arabidopsis, *WUS* expression is confined to the nucellus near the female germline (Gross‐Hardt et al., 2002; Lieber et al., 2011), whereas in *A. cherimola* it is mainly observed in the chalaza and the inner integument. In Arabidopsis, *WUS* expression in the chalaza is prevented by Class III homeodomain leucine zipper genes (*CNA*, *PHB* and *PHV*) (Yamada et al., 2016). Despite these differences, both species show highly asymmetric expression, restricted to specific regions of the ovule, and the *A. cherimola WUS* ortholog partially rescues the *wus‐1* mutant phenotype. The significance of the reversed *WUS* expression polarity in *A. cherimola* versus Arabidopsis remains unclear, but across species, the female germline develops in a polarized microenvironment (Lora et al., 2017). In *A. cherimola*, auxin distribution is found distally in the nucellus, similar to Arabidopsis (Bencivenga et al., 2012), while cytokinin distribution is proximal, close to the chalaza (Marsch‐Martínez et al., 2012; Schaller et al., 2015). Cytokinin also regulates chalazal patterning through the regulation of the polar auxin transport *PIN1* (Bencivenga et al., 2012), and *SPL/NZZ* also contributes to maintaining this polarity (Bencivenga et al., 2012). In cucumber, homologs of SPL and WUS interact (Liu et al., 2018), but whether this interaction occurs in Arabidopsis is unclear. In *A. cherimola*, their distinct expression suggests that any interaction would require protein mobility.

### *AcSPL* , *AcWUS*, and *AcWIH* expression suggests a conserved genetic pathway for MeMC initiation

*WIH1* and *WIH2* encode small proteins and act downstream of *WUS* in the same genetic pathway controlling female germline formation in Arabidopsis (Lieber et al., 2011). WIH1 and WIH3 have been included in the cysteine‐rich transmembrane module (CYSTM) family that are ubiquitous and small non‐secreted peptides (Xu et al., 2018). In *A. cherimola*, *AcWIH2* expression has been observed in MiMC and MeMC and in somatic tissues adjoining both germlines, including the anther walls and nucellus. Interestingly, the strongest *WUS* expression around the nucellus in the chalaza does not overlap with *AcWIH* expression, although weak *WUS* expression has also been observed in the distal nucellus. While the effect of the mutation of *WUS* is more restricted to the stem cells and MeMC formation (Gross‐Hardt et al., 2002; Lieber et al., 2011; Mayer et al., 1998), *wih1‐1wih2‐1* mutants show an overall twisted phenotype similar to the phenotypes of *tornado1/lopped1* (*trn1/lop1*) and *tornado2/ekeko* single mutants (Carland & McHale, 1996; Cnops et al., 2000; Lieber et al., 2011; Olmos et al., 2003). The ability of AcWUS to rescue the *wus‐1* mutant, combined with the expression patterns of *AcSPL*, *AcWUS* and *AcWIH2*, suggests a conserved genetic pathway involved in MeMC initiation between *A. cherimola* and Arabidopsis.

## CONCLUSION

In this study, we analyzed key genes involved in early female germline development in the eudicot Arabidopsis and the early‐divergent angiosperm *A. cherimola*. While some genes, such as *DMC1* and its ortholog *AcDMC1*, display similar expression patterns, most show notable differences yet remain consistently expressed within or around the germline. These differences likely reflect architectural constraints: the multi‐layered nucellus of *A. cherimola* may require broader expression domains for regulators like *AcWUS* and *AcSPL/NZZ*, whereas the single‐layered nucellus of Arabidopsis allows more localized expression. Despite these spatial shifts, core functions appear conserved, as demonstrated by the ability of *AcWUS* to partially rescue the *wus‐1* mutant. Similar expression trends in other crassinucellate eudicots (e.g., tomato, cucumber) further suggest that conserved regulators adapt to species‐specific ovule structures rather than having diverged functionally. By focusing on well‐characterized genes with clear orthology to Arabidopsis, our study provides a solid basis for comparison, rather than extending into unvalidated candidates. Future work aimed at exploring lineage‐specific regulators will be essential to uncover additional layers of diversity in germline development. Overall, our findings support a model in which conserved pathways operate within diverse ovule architectures, requiring spatial adjustments rather than fundamental changes.

## EXPERIMENTAL PROCEDURES

### Plant material

Adult trees of the *A. cherimola* cultivar Campas located in a cultivar field collection at the Institute for Mediterranean and Subtropical Horticulture ‘La Mayora’ (IHSM La Mayora‐CSIC‐UMA), Málaga, Spain, and *Arabidopsis thaliana* wild‐type ecotype Columbia‐0 and *wuschel‐1* mutant plants were used in this study. Arabidopsis plants were grown at a constant temperature of 22°C under a 16 h:8 h, light:dark cycle.

### Laser capture microdissection

Anthers and pistils of *A. cherimola* covering the earliest developmental stages were fixed in ethanol‐acetic acid (3:1, v/v), left overnight at 4°C, dehydrated in an ethanol series, embedded in Technovit 9100 (Kulzer & Co, Wehrheim, Germany), and stored at −20°C. Semithin sections of 5 μm were placed on PEN‐membrane slides (Leica Microsystems, Wetzlar, Germany), washed with acetone for 10 min, and dissected using an AS‐LMD laser microdissection system (Leica Microsystems).

We captured three tissue pools from ovules and one from stamens, obtaining 36–98 sections per sample (Figure S1). Capture efficiency was not formally quantified; however, laser settings were optimized prior to collection, and the majority of targeted sections were successfully recovered without visible contamination. RNA was extracted and amplified for library construction as described previously (Tucker, Okada, Hu, et al., 2012). RNA concentration ranged from 1205 to 4 ng μl^−1^, and integrity was assessed on a LabChip GX (RNA Pico), yielding RNA Quality Scores (RQS, RIN‐equivalent) between 3.3 and 10.0 where measurable.

Two biological replicates were obtained for whole ovules containing the MeMC, while other tissues were represented by a single pooled replicate. Because some LCM‐derived samples exhibited low RNA integrity and limited yield, we adopted a pooling strategy to maximize transcript recovery and ensure the assembly of representative full‐length contigs. This approach prioritized generating a comprehensive reference for candidate gene identification rather than precise quantitative expression profiles.

### RNA‐Seq analysis

Total RNA from LCM samples was subjected to RNA sequencing. Sequencing libraries were prepared and sequenced on an Illumina Hiseq Platform (AGRF, Australia). Reads were assembled *de novo* using previously published *Annona squamosa* RNAseq data (SRA archive SRP074402 and Gupta et al. 2015) as a reference with CLC genomics Workbench (QIAGEN, Aarhus, Denmark). Read mapping was performed using the default parameters in CLC Genomics, and transcript abundance was quantified based on transcripts per million (TPM) to normalize expression levels across samples. Additionally, the resulting contigs were assembled against the recently published *A. cherimola* reference genome (Talavera et al., 2023) using Geneious Prime (Biomatters, Auckland, New Zealand) (with default parameters).

Homologous genes between *A. cherimola* and *Arabidopsis thaliana* were identified using a reciprocal best BLAST (Basic Local Alignment Search Tool) approach. *A. cherimola* transcripts were queried against the *Arabidopsis thaliana* protein database using BLASTX, followed by a reverse BLASTP search of *Arabidopsis thaliana* protein sequences against the *A. cherimola* transcript dataset. Only reciprocal best hits with an *E*‐value threshold of 1e‐50 or better were retained as potential homologs for further analysis (Table S1). Reciprocal best BLAST (RBB) analyses between the assembled contigs and the *A. cherimola* genome were also conducted in Geneious to evaluate assembly completeness and consistency.

Gene expression patterns were analyzed using k‐means clustering to group transcripts with similar expression profiles. Clustering was performed in MeV (MultiExperiment Viewer) with *k* = 20. Gene Ontology (GO) enrichment analysis was conducted to explore functional categories enriched within specific clusters. GO annotations were obtained and analyzed using Panther (version 16) (Mi et al., 2021) and ShinyGO (Ge et al., 2020).

### Phylogenetic analysis

To confirm the identity of putative *A. cherimola* genes involved in megasporogenesis of Arabidopsis, we identified orthologous *A. cherimola* genes for a set of Arabidopsis megasporogenesis‐related genes. Using RNA‐Seq data, the following *A. cherimola* genes were identified: *AcDMC1*, *AcTAA1*, *AcKLU*, *AcSPL/NZZ*, *AcTPL*, *AcWUS*, and *AcWIH*. The protein sequences were aligned using CLUSTALX v.1.82 (Thompson et al., 1997). Their close homologs were obtained from the genome of *A. cherimola* (Talavera et al., 2023). For *A. squamosa*, homologous sequences were retrieved from the recently available genome assembly (accession: GCA_035584095.1, Peña‐Ramiréz et al., 2024) using Geneious Prime 2024.2.1 (https://www.geneious.com). In cases where multiple partial sequences were present for *A. squamosa*, a consensus sequence for each gene was generated through multiple sequence alignment using CLUSTALX v.1.82. Alignment of the WIH and SPL/NZZ proteins was manually edited using MEGA version X (Kumar et al., 2018) to remove poorly conserved regions. The remaining alignments were edited as described previously (Lora et al., 2017) using GBLOCK v.0.91b (Castresana, 2000; Talavera & Castresana, 2007). Phylogenetic protein analyses were conducted using Bayesian inference in MrBayes (Ronquist et al., 2012). The protein trees were sampled every 100 generations for varying generations depending on the dataset: 1 000 000 generations for TPL, TAA1, and SPL/NZZ; 2 100 000 generations for DMC1; 1 600 000 generations for WUS; 2 000 000 generations for KLU; and 2 750 000 generations for WIH. The first 25% of the trees of each run were discarded as burn‐in. Bayesian analysis was performed using the model of amino acid substitutions as recommended by MEGA version X (Kumar et al., 2018) that was the JTT + Invariant (I) + Gamma (G) model for the TPL, TAA1,SPL/NZZ proteins, the JTT + G model for the DMC1 and WUS proteins, the LG + G model for the KLU proteins and the WAG + G + F model for the WIH proteins.

### cDNA synthesis

Total RNA was extracted from anthers and pistils of *A. cherimola* combining the CTAB DNA extraction protocol (Doyle & Doyle, 1987) and the Qiagen RNeasy Plant Mini kit (Qiagen, Hilden, Germany). The cDNA for *in situ* hybridization and functional analyses was synthesized using the AccuPower RT/PCR PreMix (Bioneer, Daejeon, South Korea), while cDNA for RT‐qPCR analysis was synthesized using Superscript^®^ III First‐Strand Synthesis SuperMix (Invitrogen, Carlsbad, CA, USA) starting with 700 ng of RNA that was then diluted by adding 10 μl of DEPC‐treated water. PCRs were performed by standard methods using BioTaq (Bioline, London, UK) and Phusion (Thermo Scientific, St Leon‐Rot, Germany).

### *In situ* hybridization

Tissue preparation and *in situ* hybridization were performed as described previously (Mayer et al., 1998), with the following modifications. For the synthesis of antisense and sense probes, we used pBluescript II KS (+) plasmid containing a partial sequence of *AcDMC1* (JLA13), *AcTAA1* (JLA14), *AcWUS* (JLA15), *AcWIH2* (JLA19), and *AcKLU* (JLA21) that were synthesized by GenScript Biotech (Netherlands). For the antisense probes of these genes, the plasmids were linearized with *Nde*I and transcribed using T7 RNA polymerase, while for sense probes, the plasmids were linearized with *Nco*I and transcribed using T3 RNA polymerase (Table S5). For antisense and sense *AcSPL*/*NZZ*, we introduced a partial sequence of *AcSPL*/*NZZ* in antisense and sense orientations into the pGEMT‐easy vector (Promega, Madison, WI, USA), which was linearized with NcoI and transcribed using T7 RNA polymerase (Table S5). The slides of *in situ* hybridization were mounted with distilled water and photographed under differential interface contrast (DIC) using a Leica DM LB2 microscope.

### Quantitative RT‐PCR analysis

The quantitative gene expression studies were performed through a CFX96 Touch Real‐Time PCR Detection System (Bio‐Rad, Hercules, CA, USA) using 1 μl of cDNA in a 20 μl final reaction volume, following the manufacturer's instructions (Bio‐Rad, München, Germany). To identify suitable reference genes as internal controls to normalize gene expression, we tested the stability of the following *A. cherimola* genes across the four developmental stages analyzed: *Ubiquitin carrier‐like* (*AcUBCc*), *Glyceraldehyde‐3‐phosphate dehydrogenase cytosolic* (*AcGAPC*), and *Elongation factor 1‐alpha* (*AcEF1α*). Stability analysis was performed using the RefFinder web‐tool (Xie et al., 2012). *AcUBC*c was identified as the most stable reference gene and was used to normalize the expression levels (Figure S14). The means of the relative quantification (2^−ΔCT^) were calculated from a technical triplicate of three biological replicates. Primer sequences are listed in Table S6. Data were analyzed using anova, and Duncan's multiple range test (*P* ≤ 0.05) was used for means separation. Statistical analyses were performed with SPSS 12.0.

### Microscopy

To determine the developmental stages of *A. cherimola* used in the RT‐qPCR analysis, part of the anthers and pistils were fixed in 4% paraformaldehyde in PBS at pH 7.3, left overnight at 4°C, dehydrated in an acetone series, embedded in Technovit 8100 (Kulzer & Co), polymerized at 4°C, and sectioned at 2 μm. The semi‐thin sections were stained with periodic acid‐Schiff (PAS) reagent and toluidine blue (Sigma‐Aldrich) (Feder & O'Brien, 1968). For evaluating ovule development in Arabidopsis transgenic lines, ovules were fixed and cleared for light microscopy following previously published protocols (Lieber et al., 2011).

### Functional analysis

The CDS sequence of *AcWUS* was isolated using the primers described in Table S4 and based on the RNA‐Seq data. The *AcWUS* sequence was cloned into the pJET vector using the CloneJET^tm^ PCR Cloning Kit (Thermo Scientific). It was then subcloned into the previously described *pWUS:3´WUS* (MT310) (Tucker, Okada, Hu, et al., 2012) (double colon denotes fusions of promoter to expressed region hereafter) via *Sac*I and *Hind*III digestion. The resulting *pWUS:AcWUS* was further subcloned into JC30, a binary pGREENII‐0125 vector containing the 1,5 kb *WUS* terminator (Tucker, Okada, Hu, et al., 2012), via *Asc*I digestion. The final construct *pWUS:AcWUS 3´WUS* (JLA32) was introgressed into the *wus‐1* mutant using the floral dip method (Clough & Bent, 1998). The *wus‐1* mutant was identified as described previously (Lin et al., 2016).

### Subcellular localization of AcWUS using cherimoya mesophyll protoplasts

The *AcWUS* sequence was cloned into the pJET vector using the CloneJET™ PCR Cloning Kit (Thermo Scientific) and the primers described in Table S4. *AcWUS* was then subcloned into a binary pGREENII‐0125 vector containing the CaMV 35S promoter (JC179) via *Not*I digestion. The resulting *p35S:AcWUS‐YFP* (JLA53) construct was used for the subcellular localization of *AcWUS*. The protoplast isolation was performed based on Yoo et al. (2007) with the following modifications. For the enzyme solution, we prepared 20 mm of 4‐morpholine ethanesulfonic acid (MES) containing 9% (wt/vol) mannitol, 1,5% (wt/vol) cellulose onozuka R10 (Duchefa Biochemie), 0.4% (wt/vol) macerozyme R10 (Duchefa Biochemie) and 20 mm KCl. For the PEG‐calcium transfection solution, we prepared 40% (wt/vol) PEG 4000 in ddH_2_O containing 0.2 m mannitol and 100 mm CaCl_2_. The cherimoya mesophyll protoplasts were obtained from the first two leaves developed by the seedlings. For the DNA‐PEG‐calcium transfection, we mixed 200 μl of protoplasts, 10 μg of plasmid and 200 μl of PEG‐calcium transfection solution in a 2 ml Eppendorf tube and incubated at room temperature for 15 min.

## AUTHOR CONTRIBUTIONS

JL, MRT, and JIH planned and designed the research. JL performed most of the experiments, except for the transcriptome analysis, which was conducted by NJS, CM, and MRT. JL, MRT, and JIH wrote the manuscript and all authors contributed edits.

## CONFLICT OF INTEREST

The authors declare no conflict of interest.

## Supporting information


**Figure S1.** Laser capture microdissection (LCM) of developing ovule tissues in *Annona cherimola*.
**Figure S2.** Fresh plant material used for RNA extraction.
**Figure S3.** Phylogenetic tree of the RecA/RAD51 family inferred from Bayesian analysis.
**Figure S4.** *In situ* hybridization with antisense and sense probes for *Annona cherimola* genes in the anther.
**Figure S5.** *In situ* hybridization with antisense and sense probes for *Annona cherimola* genes in the ovule.
**Figure S6.** Phylogenetic tree of the TAA1/TAR family inferred from Bayesian analysis.
**Figure S7.** Phylogenetic tree of select genes from the cytochrome P450 (CYP) superfamily, including the KLU/CYP78A5 gene family, inferred from Bayesian analysis.
**Figure S8.** Phylogenetic tree of SPL/NZZ and SPL/NZZ‐like, EAR‐containing proteins (SPEARs) family, inferred from Bayesian analysis.
**Figure S9.** Phylogenetic tree of WUS and WUSCHEL‐related homeobox (WOX) proteins family, inferred from Bayesian analysis.
**Figure S10.** Phylogenetic tree of CYSTM family, inferred from Bayesian analysis.
**Figure S11.** Phylogenetic tree of TPL and TPR proteins family, inferred from Bayesian analysis.
**Figure S12.** Transient gene expression analysis of *AcWUS* in cherimoya protoplast.
**Figure S13.** Reproductive development and anther dehiscence in Arabidopsis transgenic lines containing *pWUS::AcWUS*.
**Figure S14.** Stability of *AcUBCc*, *AcGAPC* and *AcEF1α* expression across four developmental stages analyzed, using the geNorm (a), NormFinder (b), BestKeeper (c), and comparative Delta Ct (d) methods.

**Table S1.** Transcriptome assembly of *Annona cherimola*, expression values, and predicted Arabidopsis thaliana homologs.
**Table S2.** Contig sequences of the assembled transcripts.

**Table S3.** Evaluation of the reproductive development of the Arabidopsis transgenic lines analyzed in the functional study.
**Table S4.** Arabidopsis transgenic lines analyzed in the functional study.
**Table S5.** Oligonucleotide primers used in the isolation of *AcSPL/NZZ* and *AcWUS* genes of *A. cherimola* for *in situ* hybridization (*AcSPL/NZZ*) and subcellular localization and functional analysis (*AcWUS*), respectively.
**Table S6.** Oligonucleotide primers used in the RT‐qPCR.

## Data Availability

All relevant data can be found within the manuscript and its supporting materials. The sequencing data generated in this study have been deposited in the NCBI Sequence Read Archive (SRA) under BioProject ID (PRJNA1252560).
