# Supplementary material for: Comparative insights into molecular pathways influencing germline development in early‐divergent angiosperms
Source: Plant J. 2026 Jan 18;125(2):e70675. doi: 10.1111/tpj.70675 (PMC12812228; doi:10.1111/tpj.70675)
Supplement: Supplementary file 1 — Figure S1. Laser capture microdissection (LCM) of developing ovule tissues in Annona cherimola. Figure S2. Fresh plant material used for RNA extraction. Figure S3. Phylogenetic tree of the RecA/RAD51 family inferred from Bayesian analysis. Figure S4. In situ hybridization with antisense and sense probes for Annona cherimola genes in the anther. Figure S5. In situ hybridization with antisense and sense probes for Annona cherimola genes in the ovule. Figure S6. Phylogenetic tree of the TAA1/TAR family inferred from Bayesian analysis. Figure S7. Phylogenetic tree of select genes from the cytochrome P450 (CYP) superfamily, including the KLU/CYP78A5 gene family, inferred from Bayesian analysis. Figure S8. Phylogenetic tree of SPL/NZZ and SPL/NZZ‐like, EAR‐containing proteins (SPEARs) family, inferred from Bayesian analysis. Figure S9. Phylogenetic tree of WUS and WUSCHEL‐related homeobox (WOX) proteins family, inferred from Bayesian analysis. Figure S10. Phylogenetic tree of CYSTM family, inferred from Bayesian analysis. Figure S11. Phylogenetic tree of TPL and TPR proteins family, inferred from Bayesian analysis. Figure S12. Transient gene expression analysis of AcWUS in cherimoya protoplast. Figure S13. Reproductive development and anther dehiscence in Arabidopsis transgenic lines containing pWUS::AcWUS. Figure S14. Stability of AcUBCc, AcGAPC and AcEF1α expression across four developmental stages analyzed, using the geNorm (a), NormFinder (b), BestKeeper (c), and comparative Delta Ct (d) methods. [file TPJ-125-0-s002.pdf]

Comparative Insights into Molecular Pathways influencing Germline Development in early-divergent angiosperms.

Jorge Lora, Matthew R. Tucker, Neil J. Shirley, Chao Ma and José I. Hormaza

Supporting Figures S1-S14

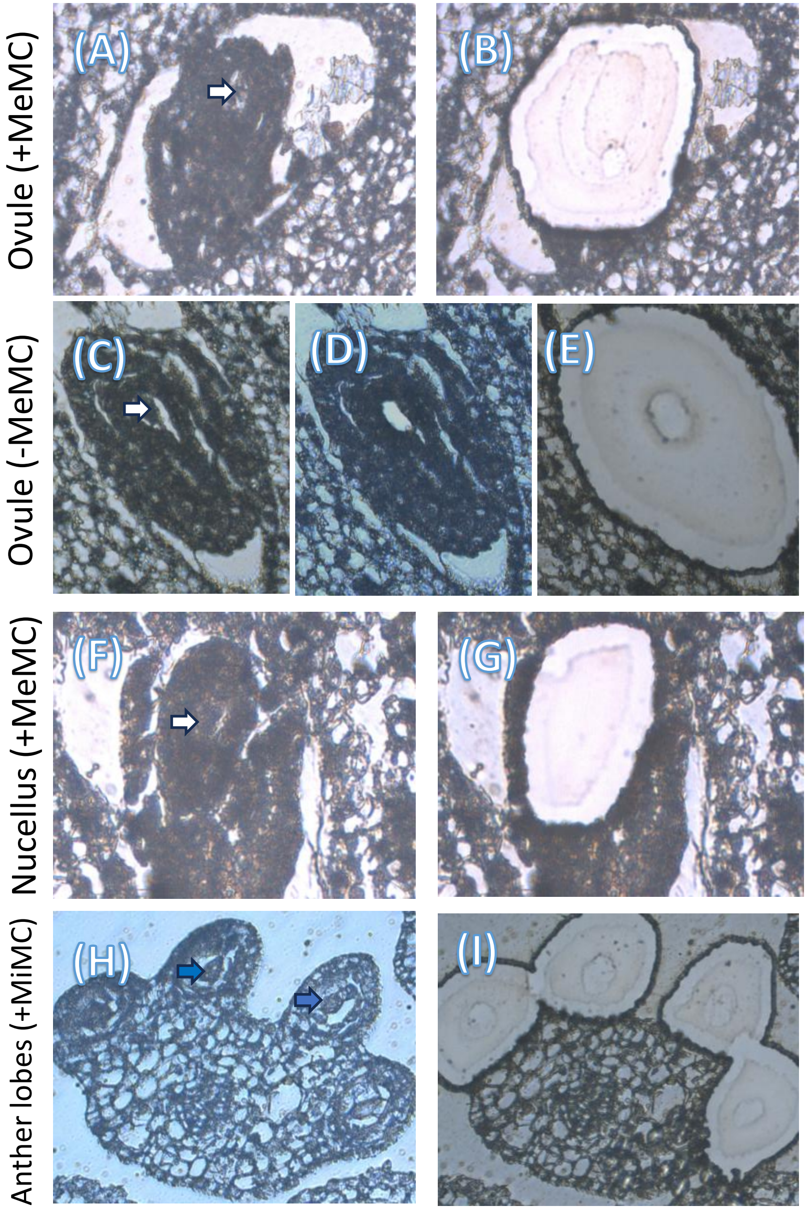

Figure S1. Laser Capture Microdissection (LCM) of developing ovule tissues in *Annona cherimola*. Representative images taken before (left) and after (right) laser capture microdissection on semi-thin section of young ovules. Four distinct tissue pools were collected: (A-B) whole ovules containing the megaspore mother cell (MeMC, white arrow), (C-E) whole ovules with the MeMC (white arrow) ablated (middle image), (F-G) the nucellus region containing the MeMC, and (H-I) the anther lobes containing the microspore mother cell (MiMC) (blue arrow).

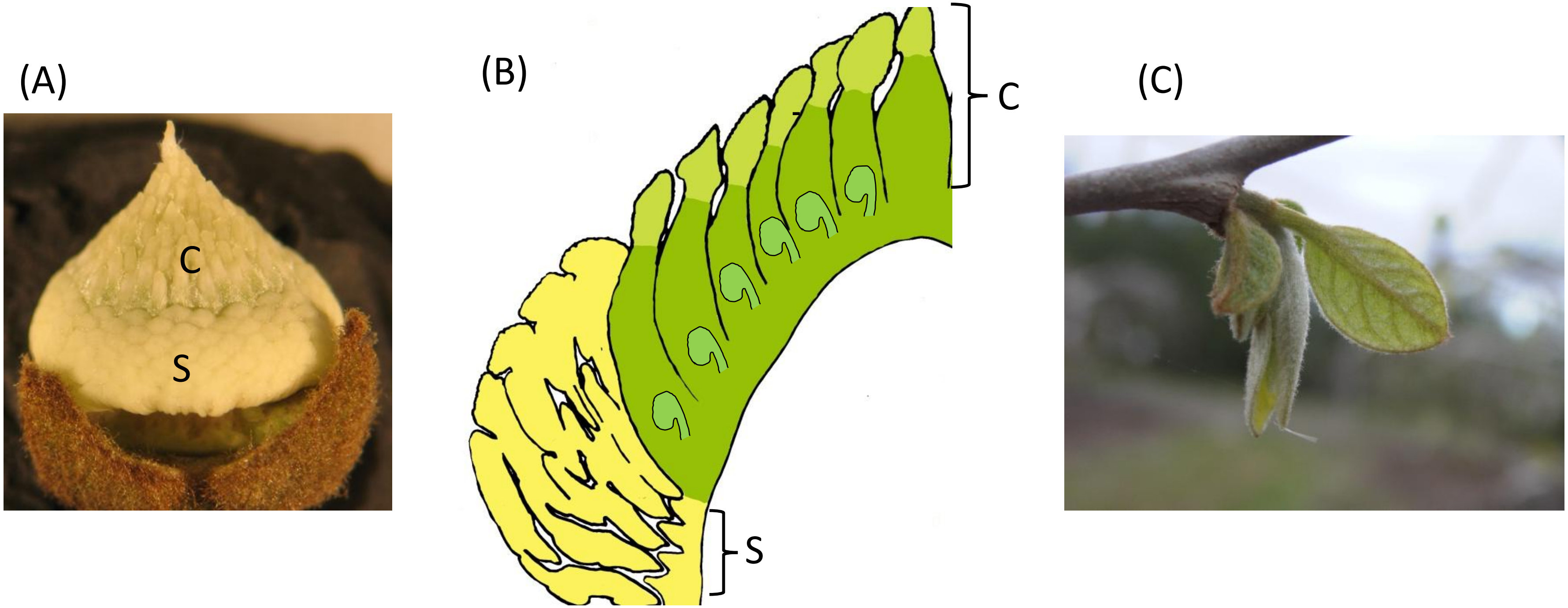

Figure S2. Fresh plant material used for RNA extraction. (A) Lateral view of a flower with petals removed. Modified from Lora et al., 2011. (B) Schematic longitudinal section of (A), highlighting the carpel in dark green and the stamens in yellow. Modified from Lora et al., 2017. (C) Young leaves. C, carpel; S, stamen.

Lora, J., Herrero, M. and Hormaza, J.I. (2011) Stigmatic receptivity in a dichogamous early-divergent angiosperm species, Annona cherimola (Annonaceae): influence of temperature and humidity. *Am. J. Bot.*, **98**, 265–274.

Lora, J., Hormaza, J.I. and Herrero, M. (2017) The Diversity of the Pollen Tube Pathway in Plants: Toward an Increasing Control by the Sporophyte. *Front. Plant Sci.*, **7**.

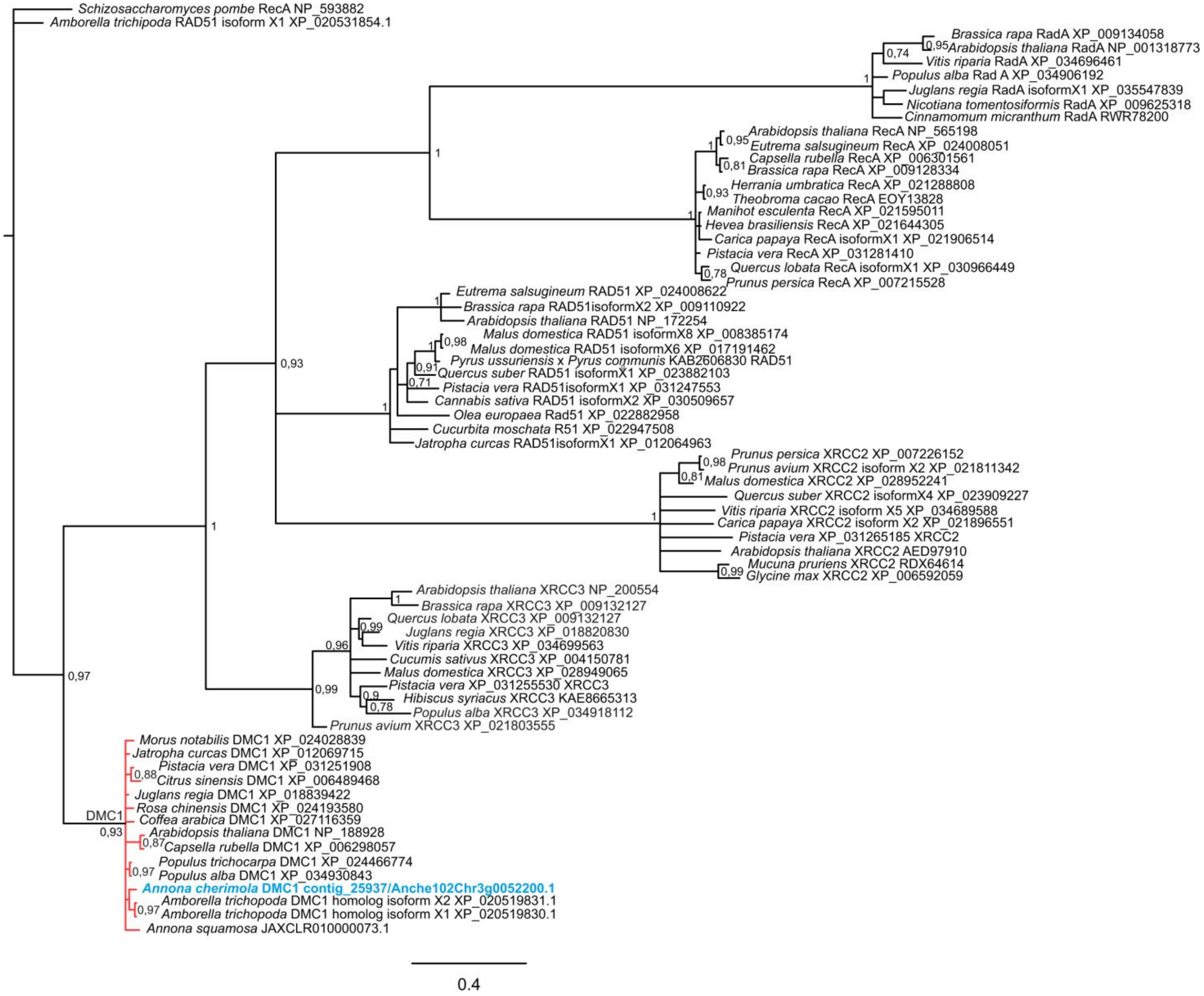

Figure S3. Phylogenetic tree of the RecA/RAD51 family inferred from Bayesian analysis. The clearly resolved clade of DMC1 orthologs is highlighted in red, with A. cherimola sequences shown in blue. Numbers adjacent to nodes indicate posterior probabilities, with only nodes >0.7 labeled. The scale bar represents 0.4 amino acid substitutions per site.

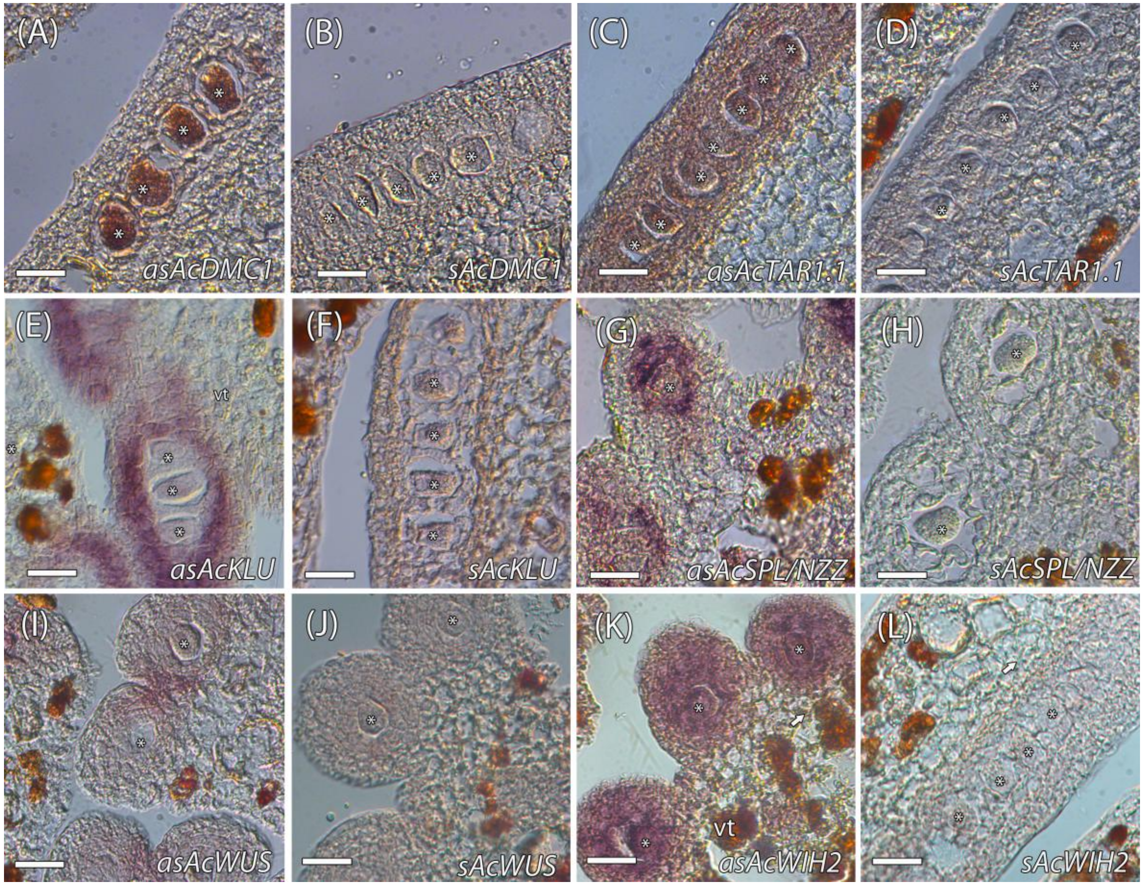

Figure S4. *In situ* hybridization with antisense and sense probes for *Annona cherimola* genes in the anther: *AcDMC1* (A-B), *AcTAR1.1* (C-D), *AcKLU*) (E-F), *AcSPL/NZZ* (G-H), *AcWUS* (I-J), and *AcWIH2* (K-L) in *A. cherimola*.

(A) Expression of antisense *AcDMC1* was observed specifically in the microspore mother cell (MiMC, indicated by asterisks).

(C) Expression of antisense *AcTAR1.1* was observed in the anther wall and MiMC (asterisks).

(E) Expression of antisense *AcKLU* was observed in the anther wall surrounding the MiMC (asterisks).

(G) Expression of antisense *AcSPL/NZZ was* observed around the MiMC (asterisks). (I) Expression of antisense *AcWUS* was observed in the stomium region.

(K) Expression of antisense *AcWIH2* was observed in the anther wall and in the MiMC.

(B, D, F, H, J, L) *In situ* hybridization using sense probes for the *A. cherimola* genes did not show any signal.

Bars = 25 µm.

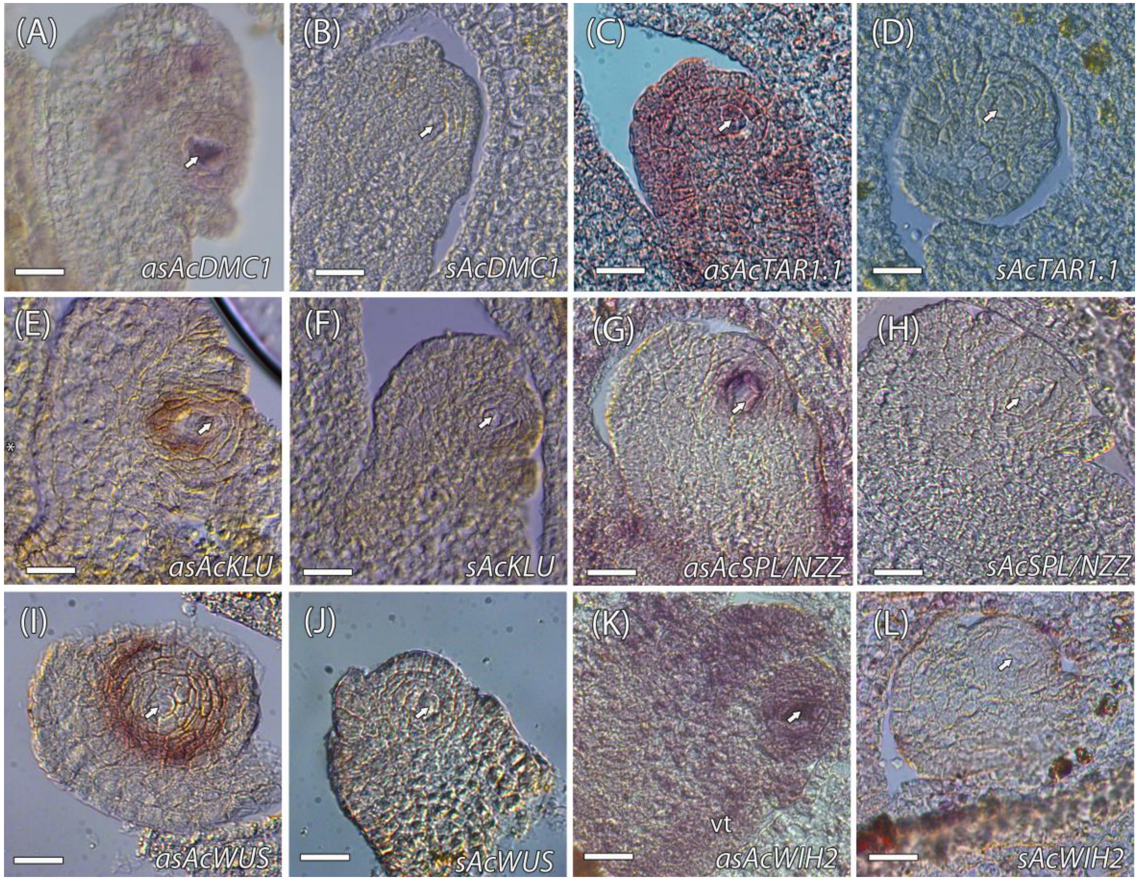

Figure S5. *In situ* hybridization with antisense and sense probes for *Annona cherimola* genes in the ovule: *AcDMC1* (A-B), *AcTAR1.1* (C-D), *AcKLU*) (E-F), *AcSPL/NZZ* (G-H), *AcWUS* (I-J), and *AcWIH2* (K-L) in *A. cherimola*.

(A) Expression of antisense *AcDMC1* was observed specifically in the megaspore mother cell (MeMC, arrow).

(C) Expression of antisense *AcTAR1.1* was observed in the ovule, including the MeMC (arrow).

(E) Expression of antisense *AcKLU* was observed in the nucellus around the MeMC (arrow).

(G) Expression of antisense *AcSPL/NZZ was* observed around the megaspore mother cell (MeMC). (I) Expression of antisense *AcWUS* was observed around the nucellus at the boundary between the inner integument and the nucellus.

(K) Expression of antisense *AcWIH2* was observed in the integuments, nucellus, and MeMC, but not in the boundary between the inner integument and the nucellus.

(B, D, F, H, J, L) *In situ* hybridization using sense probes for the *A. cherimola* genes did not show any signal.

Bars = 25 µm.

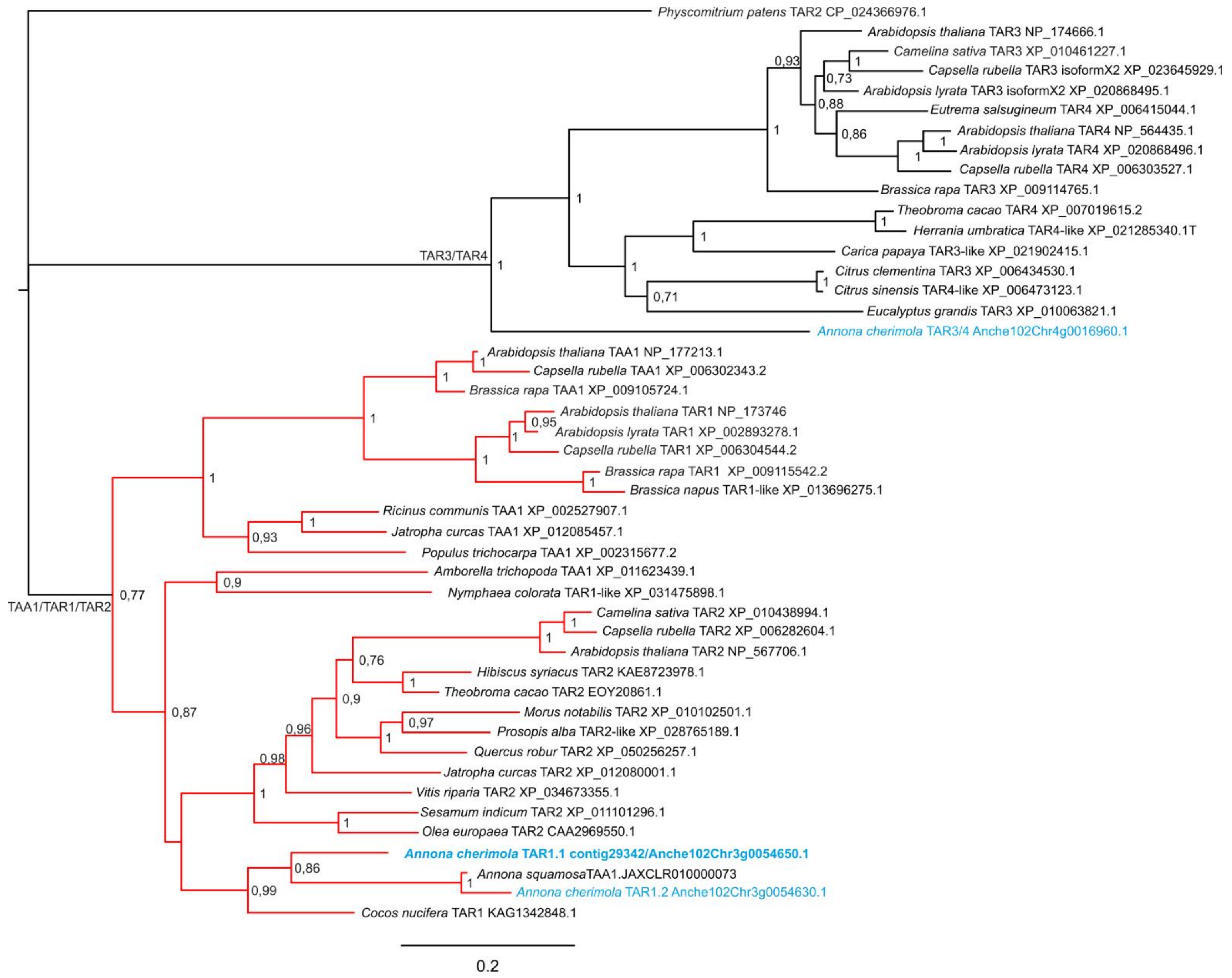

Figure S6. Phylogenetic tree of the TAA1/TAR family inferred from Bayesian analysis. The clearly resolved clade of TAA1/TAR1/TAR2 orthologs is highlighted in red, with *A. cherimola* sequences shown in blue. Numbers adjacent to nodes indicate posterior probabilities, with only nodes >0.7 labeled. The scale bar represents 0.2 amino acid substitutions per site.

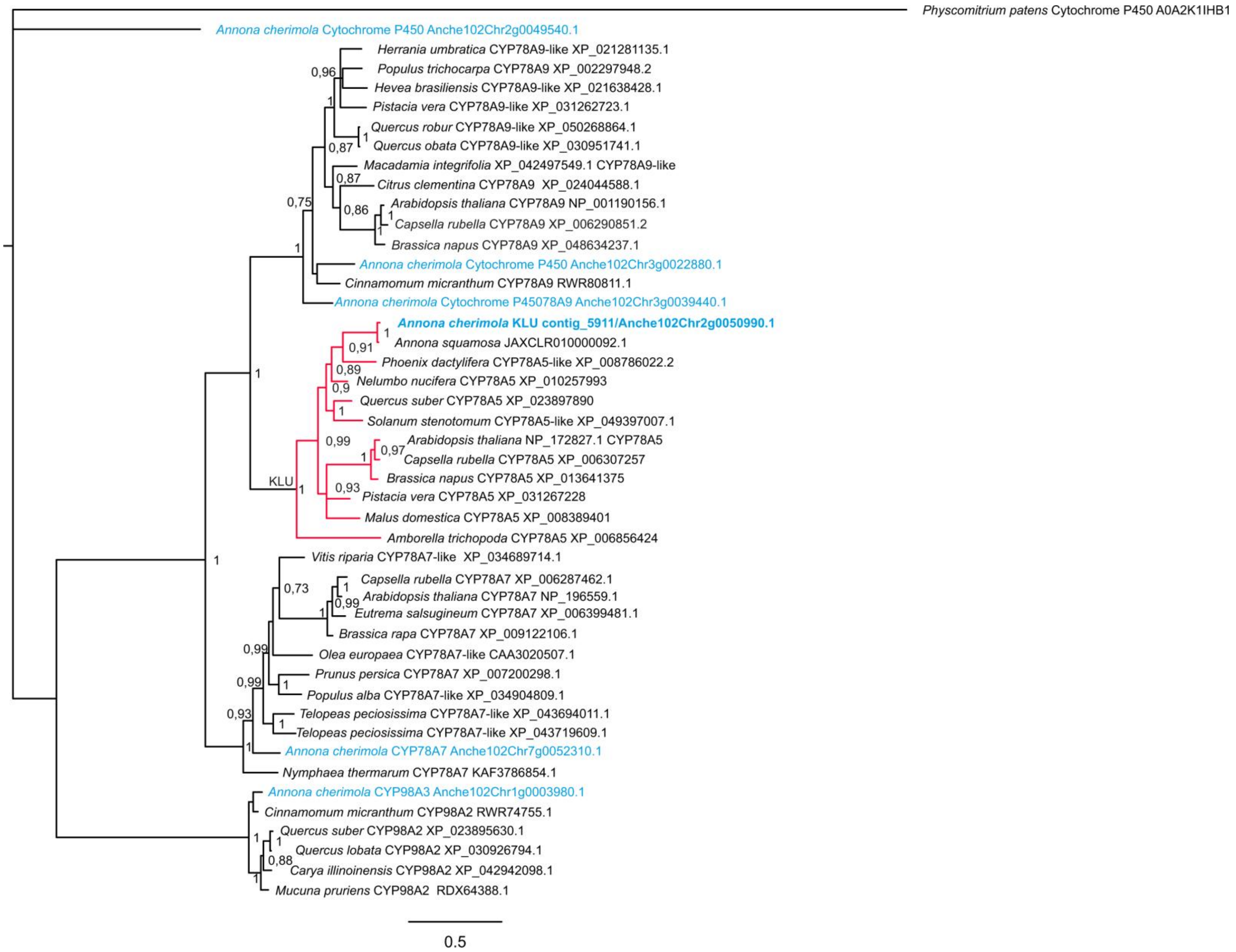

Figure S7. Phylogenetic tree of select genes from the cytochrome P450 (CYP) superfamily, including the KLU/CYP78A5 gene family, inferred from Bayesian analysis. The clearly resolved clade of KLU/CYP78A5 orthologs is highlighted in red, with *A. cherimola* sequences shown in blue. Numbers adjacent to nodes indicate posterior probabilities, with only nodes >0.7 labeled. The scale bar represents 0.5 amino acid substitutions per site.

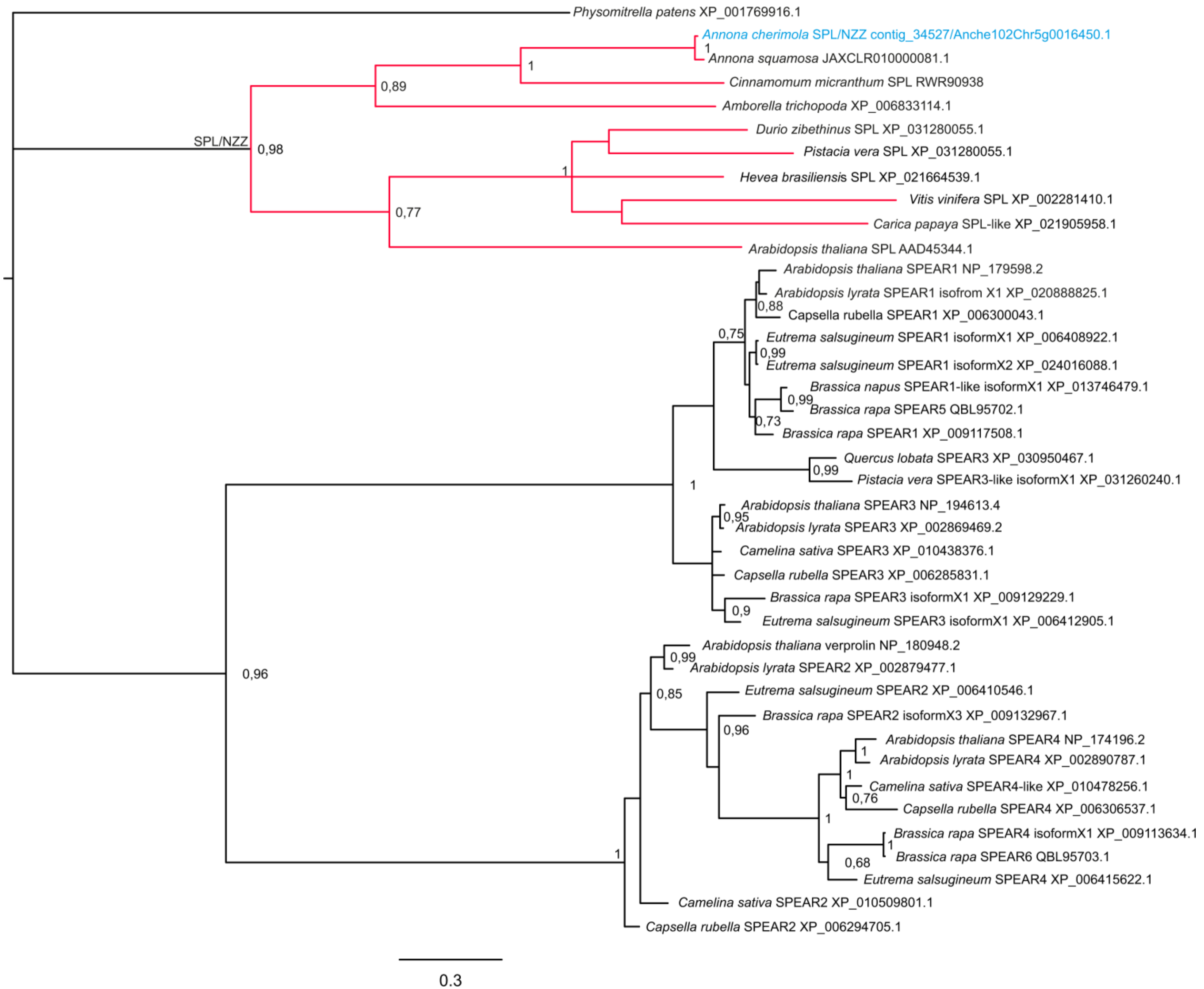

Figure S8. Phylogenetic tree of SPL/NZZ and SPL-like, EAR-containing proteins (SPEARs) family, inferred from Bayesian analysis. The clearly resolved clade of SPL orthologs is highlighted in red, with *A. cherimola* sequences shown in blue. Numbers adjacent to nodes indicate posterior probabilities, with only nodes >0.7 labeled. The scale bar represents 0.3 amino acid substitutions per site.

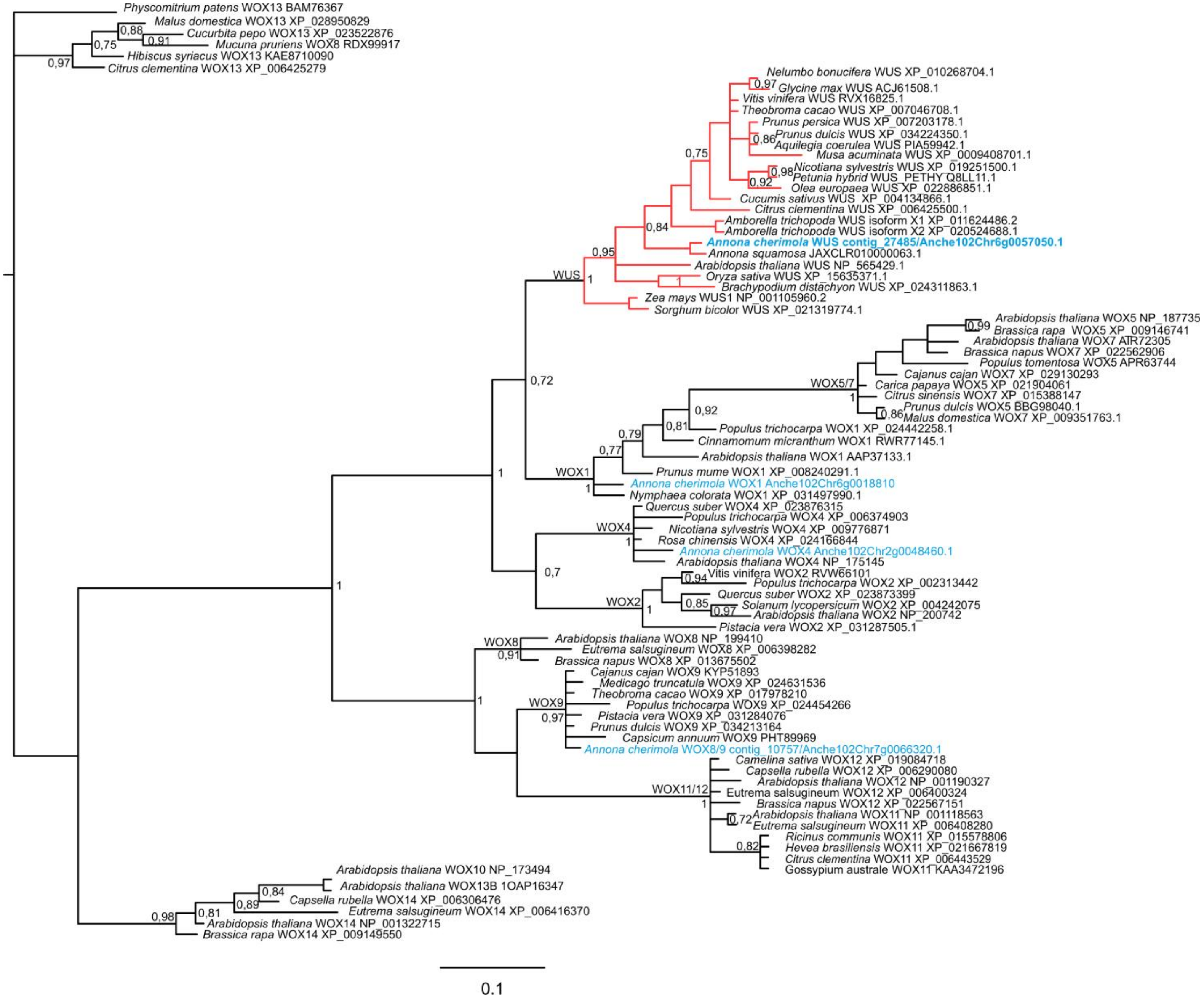

Figure S9. Phylogenetic tree of WUS and WUSCHEL-related homeobox (WOX) proteins family, inferred from Bayesian analysis. The clearly resolved clade of WUS orthologs is highlighted in red, with *A. cherimola* sequences shown in blue. Numbers adjacent to nodes indicate posterior probabilities, with only nodes >0.7 labeled. The scale bar represents 0.1 amino acid substitutions per site.

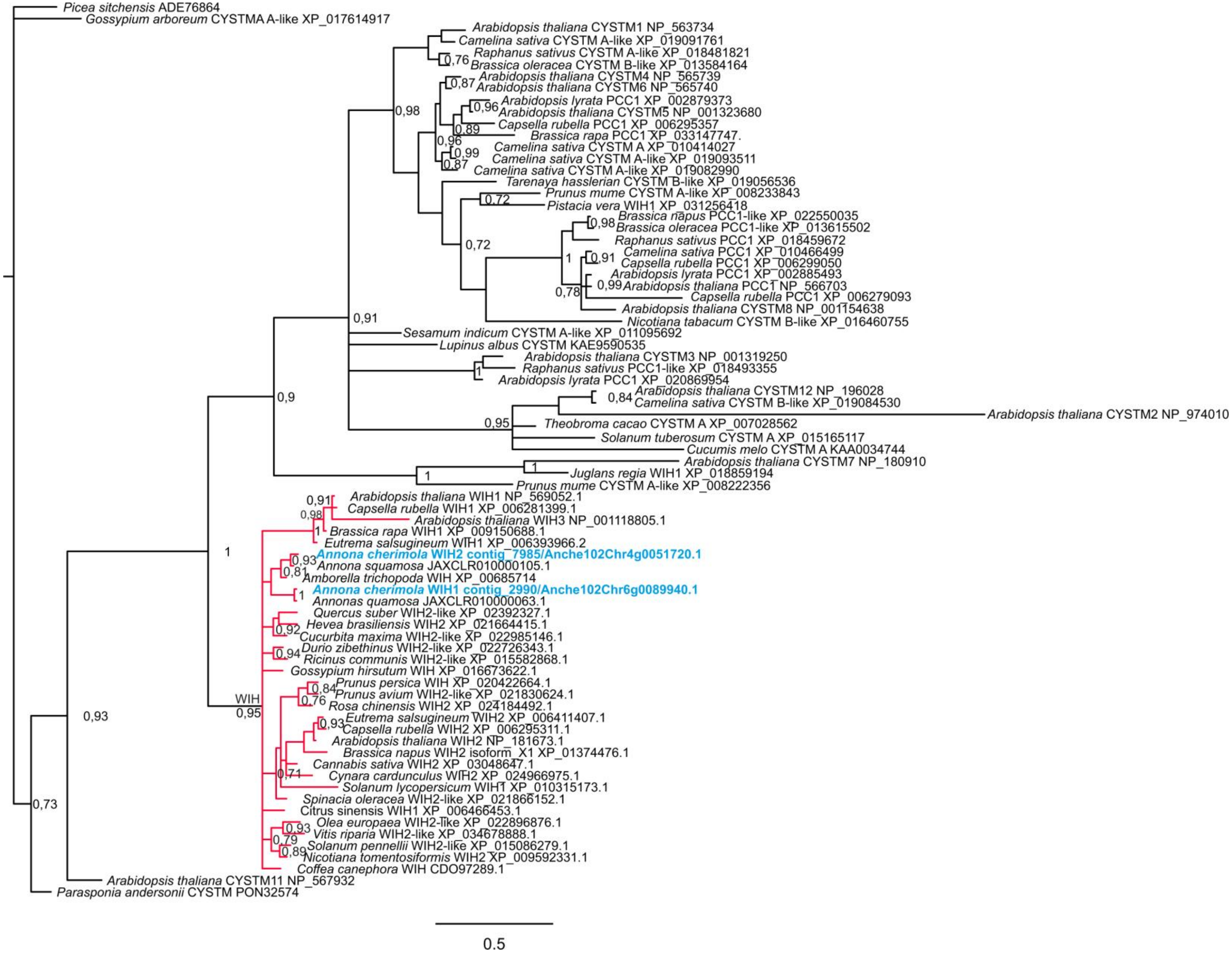

Figure S10. Phylogenetic tree of CYSTM family, inferred from Bayesian analysis. The clearly resolved clade of WIH orthologs is highlighted in red, with *A. cherimola* sequences shown in blue. Numbers adjacent to nodes indicate posterior probabilities, with only nodes >0.7 labeled. The scale bar represents 0.5 amino acid substitutions per site.

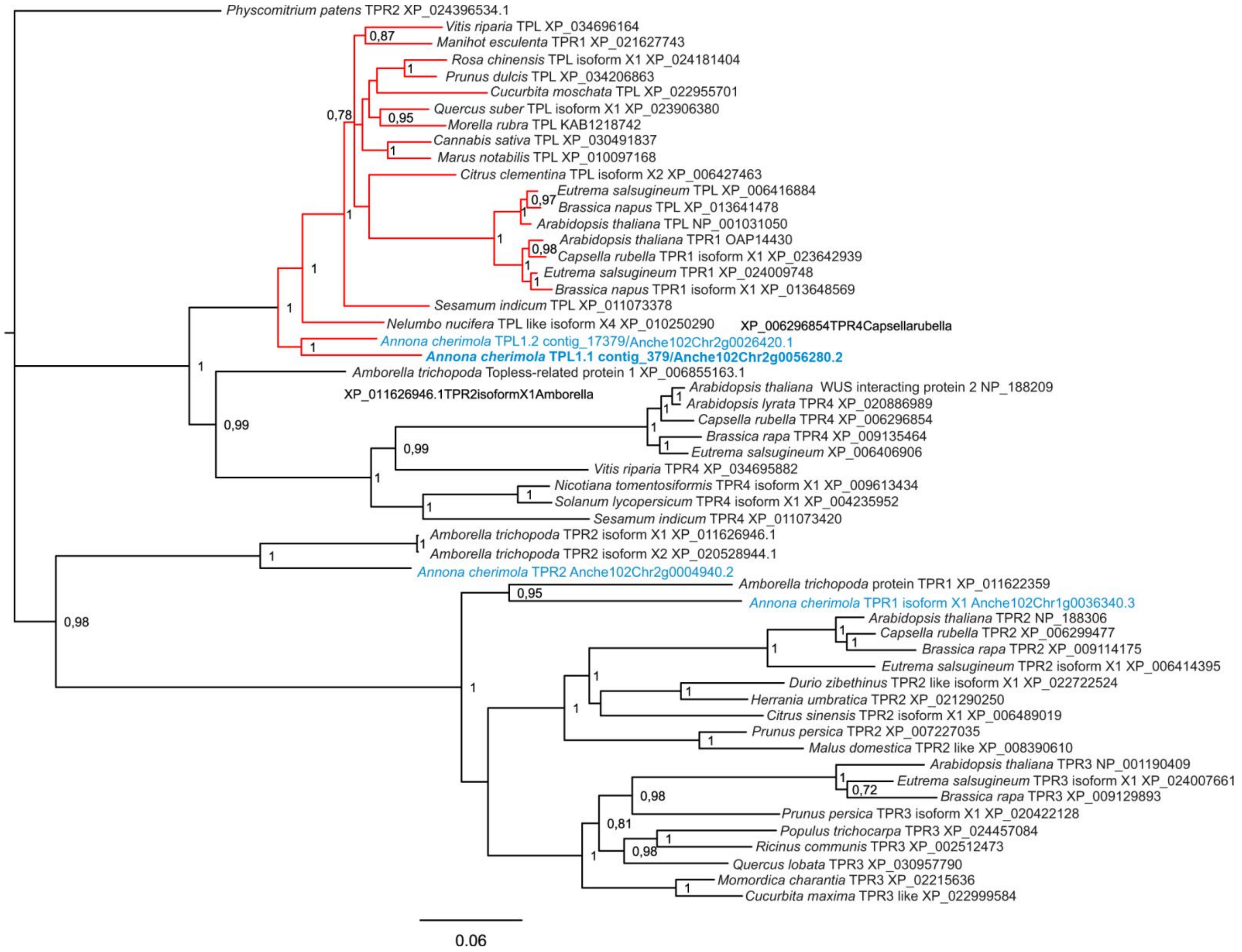

Figure S11. Phylogenetic tree of TPL and TPR proteins family, inferred from Bayesian analysis. The clearly resolved clade of TPL orthologs is highlighted in red, with *A. cherimola* sequences shown in blue. Numbers adjacent to nodes indicate posterior probabilities, with only nodes >0.7 labeled. The scale bar represents 0.06 amino acid substitutions per site.

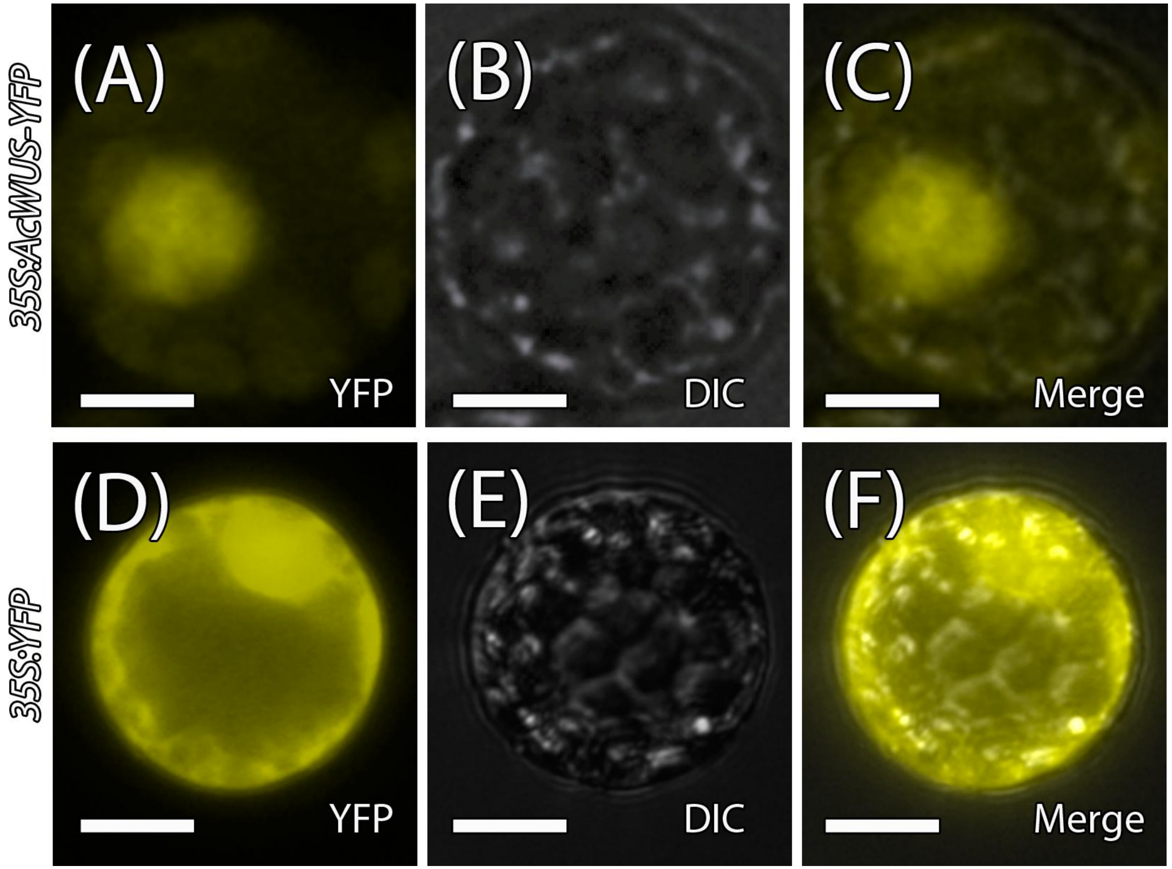

Figure S12. Transient gene expression analysis of *AcWUS* in cherimoya protoplast.
(A-C) Nuclear expression of AcWUS visualized using *35S:AcWUS-YFP*.
(D-F) Overall expression of 35S:YFP. Merged images are shown with YFP and Diferential Interference Contrast (DIC).
Bars = 10 µm.

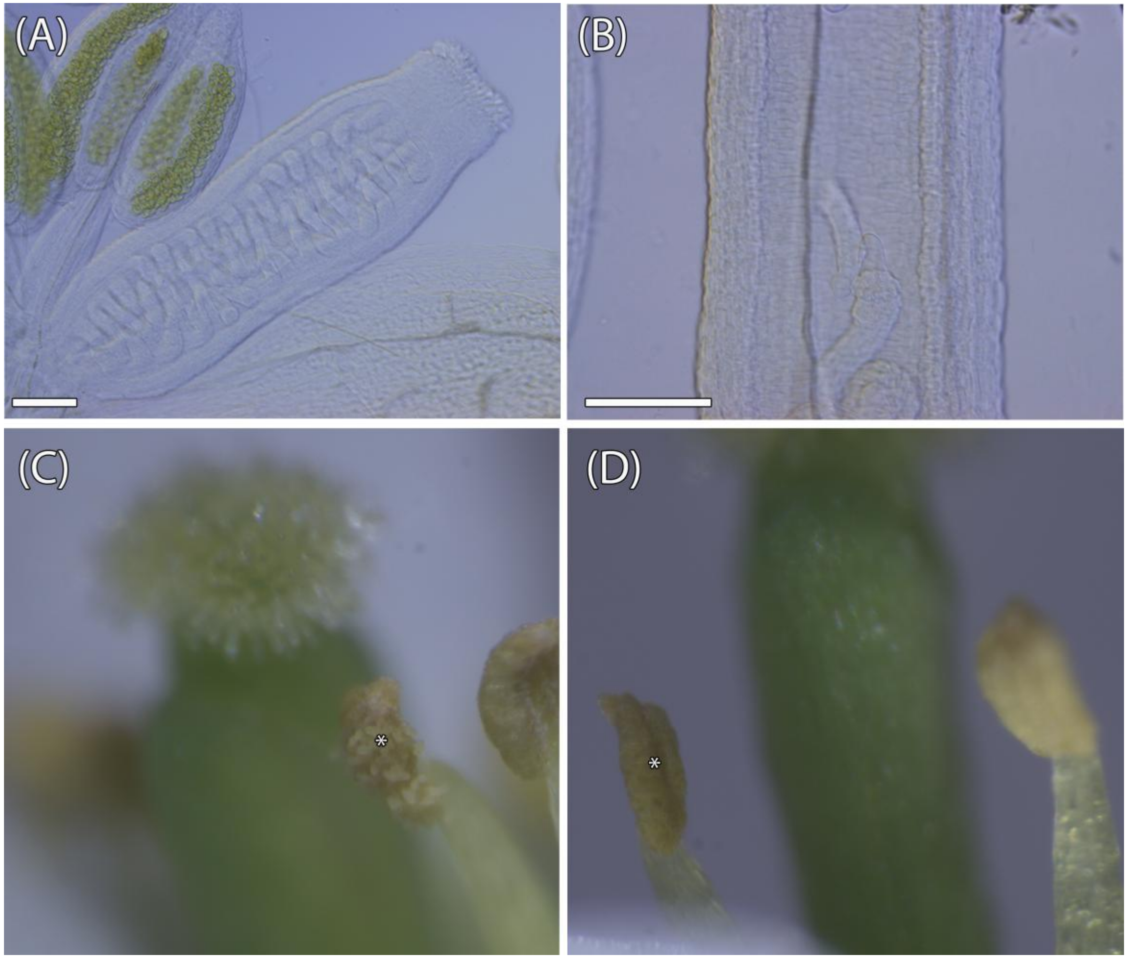

Figure S13. Reproductive development and anther dehiscence in *Arabidopsis* transgenic lines containing *pWUS:AcWUS*.
(A) Early ovule development showing the initiation of integuments in a pistil with a full complement of ovules.
(B) Gynoecium with reduced ovule number.
(C) Dehiscent anther (asterik) resembling wild-type morphology.
(D) Non-dehiscent anther (asterik) showing failure to open.

Bars = 50 µm.

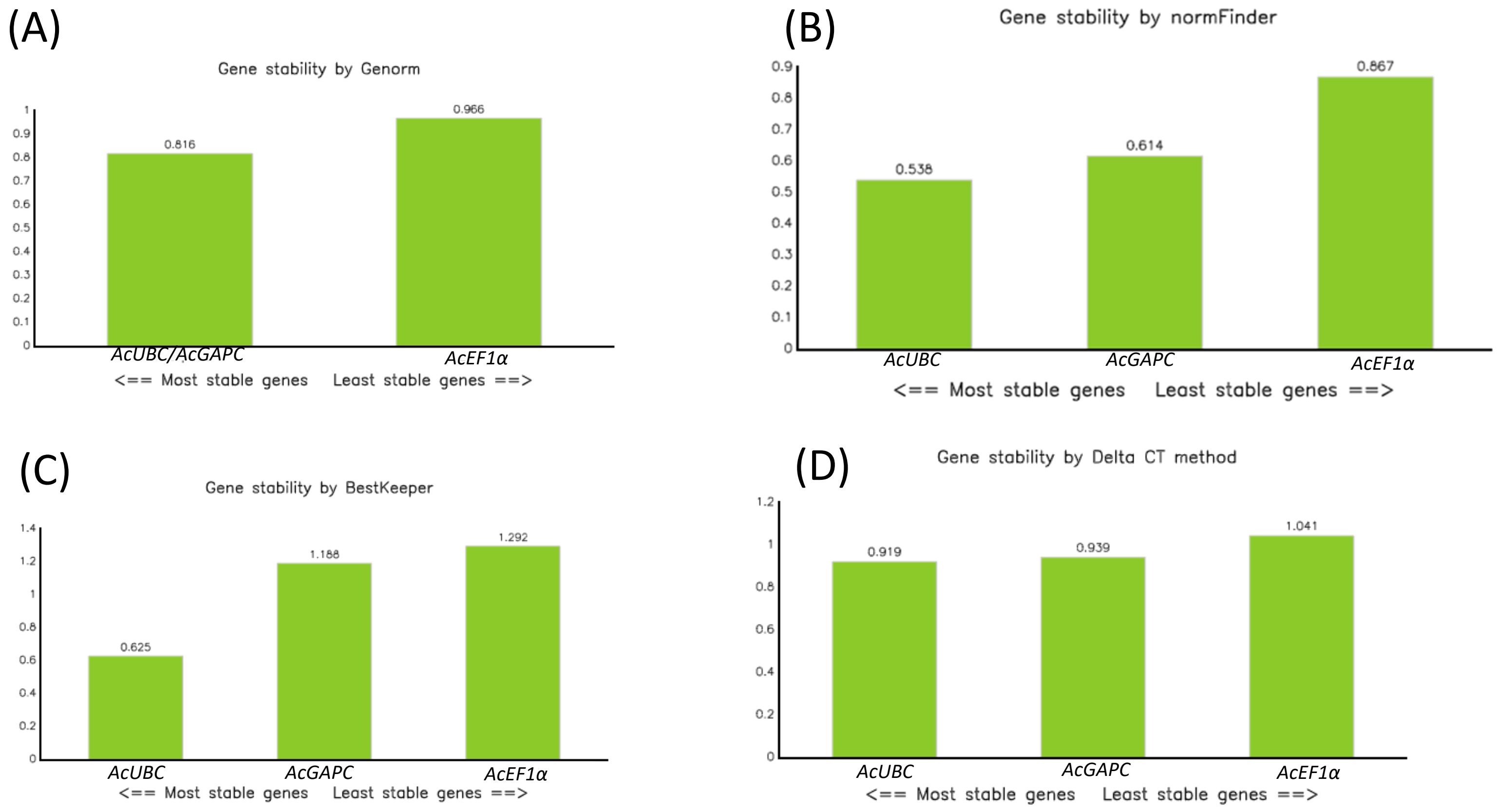

Figure S14 Stability of *AcUBCc*, *AcGAPC* and *AcEF1α* expression across four developmental stages analysed, using the geNorm (A), NormFinder (B), BestKeeper (C), and comparative Delta Ct (D) methods.
